# Inhibition of mitochondrial fission and iNOS in the dorsal vagal complex protects from overeating and weight gain

**DOI:** 10.1016/j.molmet.2020.101123

**Published:** 2020-11-20

**Authors:** Bianca Patel, Lauryn E. New, Joanne C. Griffiths, Jim Deuchars, Beatrice M. Filippi

**Affiliations:** School of Biomedical Sciences, Faculty of Biological Sciences, University of Leeds, United Kingdom

**Keywords:** Insulin resistance, DRP1, iNOS, Dorsal vagal complex-astrocytes, Mitochondrial dynamics, Food intake

## Abstract

**Objectives:**

The dorsal vagal complex (DVC) senses insulin and controls glucose homeostasis, feeding behaviour and body weight. Three-days of high-fat diet (HFD) in rats are sufficient to induce insulin resistance in the DVC and impair its ability to regulate feeding behaviour. HFD-feeding is associated with increased dynamin-related protein 1 (Drp1)-dependent mitochondrial fission in the DVC. We investigated the effects that altered Drp1 activity in the DVC has on feeding behaviour. Additionally, we aimed to uncover the molecular events and the neuronal cell populations associated with DVC insulin sensing and resistance.

**Methods:**

Eight-week-old male Sprague Dawley rats received DVC stereotactic surgery for brain infusion to facilitate the localised administration of insulin or viruses to express mutated forms of Drp1 or to knockdown inducible nitric oxide synthase (iNOS) in the NTS of the DVC. High-Fat diet feeding was used to cause insulin resistance and obesity.

**Results:**

We showed that Drp1 activation in the DVC increases weight gain in rats and Drp1 inhibition in HFD-fed rats reduced food intake, weight gain and adipose tissue. Rats expressing active Drp1 in the DVC had higher levels of iNOS and knockdown of DVC iNOS in HFD-fed rats led to a reduction of food intake, weight gain and adipose tissue. Finally, inhibiting mitochondrial fission in DVC astrocytes was sufficient to protect rats from HFD-dependent insulin resistance, hyperphagia, weight gain and fat deposition.

**Conclusion:**

We uncovered new molecular and cellular targets for brain regulation of whole-body metabolism, which could inform new strategies to combat obesity and diabetes.

## 1 Introduction

Diabetes and obesity are epidemic diseases with increasing incidence worldwide. Overnutrition is the predominant pathogenic inducer of insulin resistance, mainly caused by increased circulating levels of glucose, free fatty acids, and amino acids [1,2]. An increase in these circulating factors causes mitochondrial oxidative stress and endoplasmic reticulum (ER) stress in peripheral tissues [3] and the central nervous system (CNS) [4]. The CNS is instrumental in regulating metabolic homeostasis because it receives and processes peripheral inputs reporting the metabolic status of an individual and signals back to peripheral organs to maintain energy balance. Subtle imbalance of these homeostatic processes can lead to metabolic diseases with obesity and diabetes causing major human health problems and straining health services worldwide. Overnutrition has a substantial impact on the CNS and leads to a loss of the brain's ability to sense changes in hormone and nutrient levels. This loss dramatically affects metabolic homeostasis such that individuals have difficulty maintaining whole-body energy balance, leading to obesity and diabetes [5,6]. Restoring the brain's ability to modulate metabolic functions could be important to prevent the negative outcomes of obesity and diabetes.

Insulin is one of the major humoral signals that modulates energy balance. Deletion of the insulin receptor in the brain (NIR knockout mice) led to increased body fat and insulin resistance [7]. The hypothalamus, located at the lateral boundaries of the third ventricle, is one of the major areas of the brain responsible for regulating energy homeostasis in response to changes in hormonal and nutrient levels [5]. Intracerebroventricular (ICV) injection of insulin in the mediobasal hypothalamus (MBH) decreased hepatic glucose production (HGP) [8,9], and selective decrease in insulin receptor levels in the hypothalamus resulted in hyperphagia and increased fat mass [10]. In rodents, the dorsal vagal complex (DVC) of the brain is another important regulator of glucose metabolism [11] and food intake [12]. In particular, the nucleus of the solitary tract (NTS) in the DVC senses insulin and triggers a neuronal relay to decrease HGP and food intake [11,12]. Interestingly, directly targeting the brain by intranasal delivery of insulin lowers blood glucose levels and decreases food intake in non-obese and non-diabetic humans [13,14]. This finding suggests that insulin action in the brain may be clinically relevant in the treatment of individuals who are obese and diabetic and mandates further research to fully understand the mechanisms of brain insulin signalling in animal models of obesity and diabetes.

Rodents lose the ability to sense both MBH and DVC insulin and regulate HGP and food intake [11,12,15] after 3 days on a high fat diet (HFD)**.** The development of insulin resistance in the brain was associated with increased inflammation and ER stress [16,17]. HFD, obesity, and insulin resistance in the brain have also been associated with mitochondrial dysfunction [18,19]. Mitochondria are highly dynamic organelles that change morphology through fission and fusion to meet energy demands [20]. When cellular energy demands are high, mitochondrial fusion is one way to produce more ATP [21,22]. Conversely, when the cell is in excess of energy, there is an increase in mitochondrial fragmentation (fission) that causes decreased mitochondrial activity and mitophagy [21,22]. These 2 opposing processes are regulated by proteins, namely, mitofusin 1 and 2 (Mfn-1 and -2) and optic atrophy (Opa1) that regulate mitochondrial fusion, and dynamin-related protein 1 (Drp1) and fission protein 1 (Fis1) that regulate fission. A fine balance of these processes is necessary to maintain mitochondria functionality [21].

In adipose tissue, mitochondrial dysfunction increases oxidative stress, leading to an increase in fat oxidation and lipid accumulation, which is associated with insulin resistance [21]. In particular, enhanced mitochondrial fission has been associated with diet-induced obesity and insulin resistance in different tissues. An increase in active Drp1 caused high levels of mitochondrial fission in skeletal muscle, and this response was associated with diet-induced obesity and insulin resistance [20]. In addition, deletion of Drp1 in the liver prevented mice from developing diet-induced insulin resistance [23].

Mitochondrial dynamics also play a pivotal role in how neuronal cells respond to hormonal changes. In POMC and AgRP neurones the knockdown of proteins involved in the regulation of mitochondrial dynamics can alter feeding behaviour and glucose metabolism [19,[24], [25], [26]], and the glucose-sensing neurones of the ventromedial hypothalamus change mitochondrial morphology and their firing rates in response to changes in glucose levels [27]. Diet-induced alteration of mitochondrial dynamics also occurs in extra hypothalamic regions. Indeed, HFD-dependent insulin resistance in the DVC is caused by increased mitochondrial fission through activation of Drp1 [28]. Direct injection of chemical or molecular inhibitors of Drp1 into the DVC restored the ability of insulin to regulate glucose metabolism in HFD-fed rats [28]. Conversely, direct injection of an adenovirus expressing active Drp1 in the DVC caused insulin resistance in rats fed a regular chow (RC) diet [28] and prevented DVC insulin from lowering HGP. However, whether the changes in mitochondrial fission in DVC neurones affects food intake and body weight requires further research.

This biological question is important because how HFD-dependent activation of Drp1 and mitochondrial fission cause insulin resistance remains unclear. However, there is an increase in inducible nitric oxide synthase (iNOS) levels in the DVC of HFD-fed rats and of RC-fed rats overexpressing active Drp1 [28]. Studies in rodents have suggested that increased iNOS levels cause insulin resistance in muscle of diet-induced obese and genetically obese mice [29], and S-nitrosylation and activation of ER stress-related proteins in the liver of HFD-fed rats altered metabolic functions [30]. Furthermore, in the hypothalamus, increased iNOS levels triggered insulin resistance and obesity [31]. Therefore, we investigated whether changes in iNOS levels in the DVC affect food intake and body weight.

Aberrant levels of iNOS in astrocytes lead to astrogliosis and have been shown to increase levels of neuroinflammation and neurotoxicity [32], suggesting that astrocytes may be involved in the link between HFD-induced elevation in iNOS levels and insulin resistance. Hypothalamic astrocyte-dependent inflammation (astrogliosis) was observed in diet-induced or genetically modified models of obesity; however, the exact mechanism of this is not well understood [33,34]. Whether changes in mitochondrial dynamics and iNOS levels in astrocytes are involved in the development of DVC insulin resistance requires further investigation.

In this study, we show that changes in mitochondrial dynamics in the NTS of the DVC affect insulin sensitivity, food intake, body weight, and fat deposition. We demonstrate that increasing mitochondrial fission by activating Drp1 in the NTS causes insulin resistance, hyperphagia, and body weight gain. Conversely, inhibiting Drp1 to decrease mitochondrial fission protects from HFD-depended insulin resistance and decreases food intake and body weight gain. We also show that HFD and activation of Drp1 increase iNOS levels in the DVC, and is sufficient to knock down iNOS to protect from HFD-dependent development of insulin resistance. Finally, for the first time, we show that inhibiting mitochondrial fission in astrocytes of the NTS is sufficient to protect rodents from developing HFD-dependent insulin resistance, and also results in a decreased food intake, body weight gain, and fat deposition.

The brain is the central player in maintaining energy balance; alteration of the brain's ability to maintain metabolic homeostasis is strictly correlated with the development of obesity. An improved understanding of the brain regions involved in controlling metabolic functions, and the molecular mechanisms that alter these functions, is the basis for developing new strategies to fight the obesity epidemic.

## 2 Material and methods

### 2.1 Animals

Nine-week-old male Sprague–Dawley (SD) rats weighing between 270 and 300 g (Charles River Laboratories) were used in line with the United Kingdom animals (Scientific Procedures) Act 1986 and ethical standards set by the University of Leeds Ethical Review Committee. Animals were housed individually and maintained on a 12-hour light–dark cycle with access to either RC or HFD and water ad libitum.

Rats were stereotactically implanted with a bilateral catheter targeting the NTS within the DVC: 0.0 mm on occipital crest, 0.4 mm lateral to the midline, and 7.9 mm below skull surface [28] on day 0. On day 0, a lentiviral system was used to deliver ShRNA to knockdown of iNOS (shiNOS) or a control scramble ShRNA (shControl; Santa Cruz Biotechnology, sc-29417-V and sc-108080, respectively), or on day 1, an adenoviral system was used to deliver either a constitutively active form of Drp1 (Drp1-S637A), a catalytically inactive form of Drp1 (Drp1-K38A), or a control of GFP expressed under CMV [28] or GFAP promoters.

In the first cohort, animals expressing Drp1-S367A or GFP were given RC. In the second and third cohort, animals expressing Drp1-K38A and GFP or shiNOS and shControl, were given RC for 3 days post-surgery and then were put on HFD. The diet composition of RC (3.93 kcal/g) was 20.5% protein, 7.2% fat, 3.5% ash, and 61.6% carbohydrate (Dates and group, F4031), and that of the HFD (5.51 kcal/g) was 20.5% protein, 36% fat, 3.5% ash, and 36.2% carbohydrate (Dates and group, F3282). Animals were sacrificed on day 16 and epididymal, retroperitoneal, and visceral fat were collected and weighed. The DVC was collected for western blot analysis or immunohistochemistry. Bromophenol blue was used to confirm that the NTS of the DVC region was successfully targeted in surgery.

### 2.2 Acute feeding study

On days 8 and 14 after viral injection (day 1), an acute feeding study was performed. On the morning of the study, animals were fasted for 7 h before the experiment. At 4 pm, animals were infused bilaterally with 2 mU/μl insulin or a vehicle into the DVC of the brain: 0.2 μl was infused over 5 min (0.04 μl/min). Food was returned after infusion. Food intake was measured every 30 min for 4 h and then again at 12, 24, and 36 h. Data are presented as the average of days 8 and 14.

### 2.3 Production of recombinant adenoviruses

The preparation of recombinant adenoviruses expressing FLAG-Drp1K38A, FLAG-Drp1S637A, and GFP from the CMV promoter were as previously described [28] by using the RAPAd CMV Adenoviral Expression system (Cell Biolabs, Inc. San Diego, CA, USA.), following the manufacturer's instructions. To produce vectors expressing these genes under the GFAP promoter, the CMV promoter gene was removed from the pacAD5 CMV shuttle vectors and replaced with a rat GFAP promoter gene. Cloning was conducted by using standard protocols, and DNA was extracted and purified by using Zymo Research DNA kits (Cambridge Bioscience). The recombinant adenoviruses were amplified in HEK293 AD cells and then purified by using a sucrose-based method [35]. Briefly, virus-containing media was centrifuged at 3000 rpm for 5 min and passed through a 0.4 μm filter. The media was then loaded onto 10% Sucrose/TENS (50 mM Tris–Hcl pH 7.4, 100 mMNacl, 0.5 mM EDTA) in a 4:1v/v ratio and centrifuged at 10,000 rpm for 4 h at 4 °C. The resulting virus pellet was resuspended in PBS at 4 °C overnight and the purified virus was titrated using a DAB assay (Adenovirus Monoclonal Antibody (E28H) MA17001 Invitrogen, Vector Lab SK-4100 DAB kit). The titres of the adenoviruses injected in the animals were between 9.5 × 10^8^ pfu/ml and 1.5 × 10^9^ pfu/ml.

### 2.4 Obese model

Six-week-old male SD rats weighing between (170 and 190 g) were used: from day 0, these animals were subjected to HFD or an RC for 28 days. The 28-day HFD-feeding protocol caused insulin resistance, obesity (∼10–15% increase in body weight compared with RC-fed rats), and increased basal insulin and blood glucose levels [[36], [37], [38]]. On day 28, animals were implanted with a bilateral catheter targeting the NTS within the DVC. The animals were split into 2 cohorts: the first cohort was injected with Drp1-K38A or GFP, and the second cohort was injected with shiNOS or shControl. Animals were subjected to the acute feeding study on days 37 and 41, applying the aforementioned protocol.

### 2.5 Nitrate measurement

Nitrate levels in DVC brain wedges were measured by using the Nitrate Assay Kit from BioVision (K544), according to the manufacturer's instructions. In brief, DVC wedges were weighed and homogenised in nitrate assay buffer. Half of the homogenate was then incubated with Griess reagents 1 and 2 while the other half was incubated with only Griess reagent 1 to obtain a blank for each sample. Each blank value was then subtracted from its corresponding sample before calculating the nitrate levels.

### 2.6 S-nitrosylation TMT-assay

Nitrosylated proteins were detected by using a tandem mass tag switch method [39]. Neuronal cell line, PC12 were infected for 48 h with Drp1-S637A, Drp1-K38A, or GFP-expressing adenovirus. Cell lysates were lysed with HENS buffer (ThermoScientific 90,106); GFP lysates were also treated with either glutathione or S-nitrosoglutathione as negative or positive controls, respectively, and left for 1 h. A BCA protein assay was used to make up lysates to 1 μg/μl in HENS buffer. Unmodified cysteines were then blocked by using sulfhydryl-reactive compound (MMTS). S-nitrosylated cysteines were then reduced with sodium ascorbate and specifically labelled with iodoTMTzero. Nitrosylated proteins were determined by SDS page.

### 2.7 Western blotting

Tissue Samples, PC12 cells, or DI-TNC1 astrocyte cells were lysed in lysis buffer (50 mM Tris-Hcl pH 7.5, 1mM EGTA, 1mM EDTA, 1% (w/v) NP-40, 1mM sodium orthovanadate, 50 mM sodium fluoride, 5mM sodium pyrophosphate, 0.27M sucrose, 1μM DTT and Pierce Protease Inhibitor Tablets), using a homogeniser on ice. Samples were centrifuged at 12,000 RPM for 15 min at 4° supernatants were collected and protein concentration was determined by using a Bradford Assay. Proteins were separated by using SDS page (8 or 10%) and transferred at 4 °C for 3 h. Membranes were blocked in 5% BSA in TBST, and the primary antibodies Sigma Anti-FLAG M2 F1804 (1:3,000); Aviva System Biology GFP OAE00007 (1:20,000); Abcam Anti-iNOS ab15323 (1:50); Cell Signalling Technology Phospho-PERK 3179 (1:1,000); Cell Signalling Technology Total-PERK 3192 (1:1,000); Cell Signalling Technology β-Actin 3700 (1:50,000); Invitrogen Anti-TMT 90075 (1:1000) and Abcam Anti-Nitryl tyrosine ab42789 (1:500) were left to incubate overnight at 4 °C. Membranes were imaged by using ECL (BioRad Clarity) or (BioRad ChemiDoc™ MP Imaging System). Protein levels were analysed with ImageJ (Fiji).

### 2.8 Immunofluorescence (IF)

Animals were perfused at the end of the experiment with 4% paraformaldehyde. The brainstem area containing the DVC was taken and frozen in cryoprotectant; 25 μm sections were cut using a cryostat. Sections were labelled for FLAG by using Sigma Anti-FLAG M2 F1804 (1:500) and co-stained with markers for neurones (NeuN, 1:2000, Millipore ABN90), astrocytes (GFAP, 1:1000, Abcam ab7260), oligodendrocytes (PanQKI, 1:100, Neuromab 75–168), or microglia (Iba1, 1:1000, Wako 019–19741). To stain iNOS abcam, anti-iNOS ab15323 (1:250) was used. Sections were mounted using vectasheild plus 4’,6-diamindino-2-phenylindole (DAPI) to visualise nuclei. Images were taken using a Zeiss LSM880 upright confocal laser scanning microscope.

### 2.9 IF quantification

iNOS intensity staining was quantified with Fiji, by randomly selecting 4 different areas for each picture and measuring the average intensity. For co-localisation studies, images were processed and exported by using ZEN software; figures are presented as a single plane image. Images were counted by using 3 random tiles of each slice; 3 slices were used per rodent; an average of all counts was taken and demonstrated by quantification graphs.

### 2.10 Mitochondria morphology

Single multitest 8-well glass slides (MP Biomedicals) were placed in the bottom of sterile 10 cm petri dishes. A suspension of HEK293AD cells at approximately 5 × 10^4^ cells/ml was prepared, and 25 ml of the cell suspension was added to each dish and incubated in a humid atmosphere of 5% CO_2_/air for 24 h. HEK293AD cells at 80% confluency were then infected with an adenovirus expressing either GFP, Drp1-S637A-FLAG, or Drp1-K38A-FLAG at an MOI of 5. After 24 h, MitoTracker Red CMXRos (ThermoScientific M7512) was added to the media to provide a final concentration of 0.5 μM and incubated at 37 °C for 45 min. Cells were then washed with PBS and fixed in 4% PFA for 30 min at RT. After fixation, multitest slides were removed from petri dishes and stored in humidified chambers for the remaining steps. Cells were washed in PBS and blocked for 1 h at RT with 10% donkey serum, 0.5% BSA in PBS. Cells were stained with mouse monoclonal anti-FLAG (1:800 in 0.1% PBST) overnight at 4 °C followed by secondary antibody detection using an Alexa Fluor 488 conjugated donkey anti-mouse IgG (1:800) for 1 h at RT. Cells were washed 3 x in PBS and allowed to dry slightly before adding vectashield plus DAPI mounting medium and sealing with a coverslip.

Cells were imaged using a Zeiss LSM880 upright confocal laser scanning microscope and were processed in Fiji, according to methods described by Merrill et al. (2017), to highlight mitochondria for morphological analysis [40]. The “analyse particles function” in Fiji was then used to analyse mitochondrial morphometry. Mitochondria AR was estimated by dividing the major axis by the minor axis, reflecting the length to width ratio. Form factor was calculated as perimeter^2^/4π·area.

### 2.11 Statistical analysis

All data are expressed as mean ± SEM. Data were analysed by using GraphPad Prism 7 software. A significant difference was determined by using multiple T-tests, one-way ANOVA (post-doc test: Sidak), or a two-way ANOVA (post-doc test: Turkey). N refers to the number of animals used. P < 0.05 was considered to be statistically significant. Significance was defined by (∗) P < 0.05; (∗∗) P < 0.01; (∗∗∗) P < 0.001; (∗∗∗∗) P < 0.0001.

## 3 Results

### 3.1 Higher mitochondrial fission in the DVC causes insulin resistance, hyperphagia, and body weight gain

**Figure 1:**
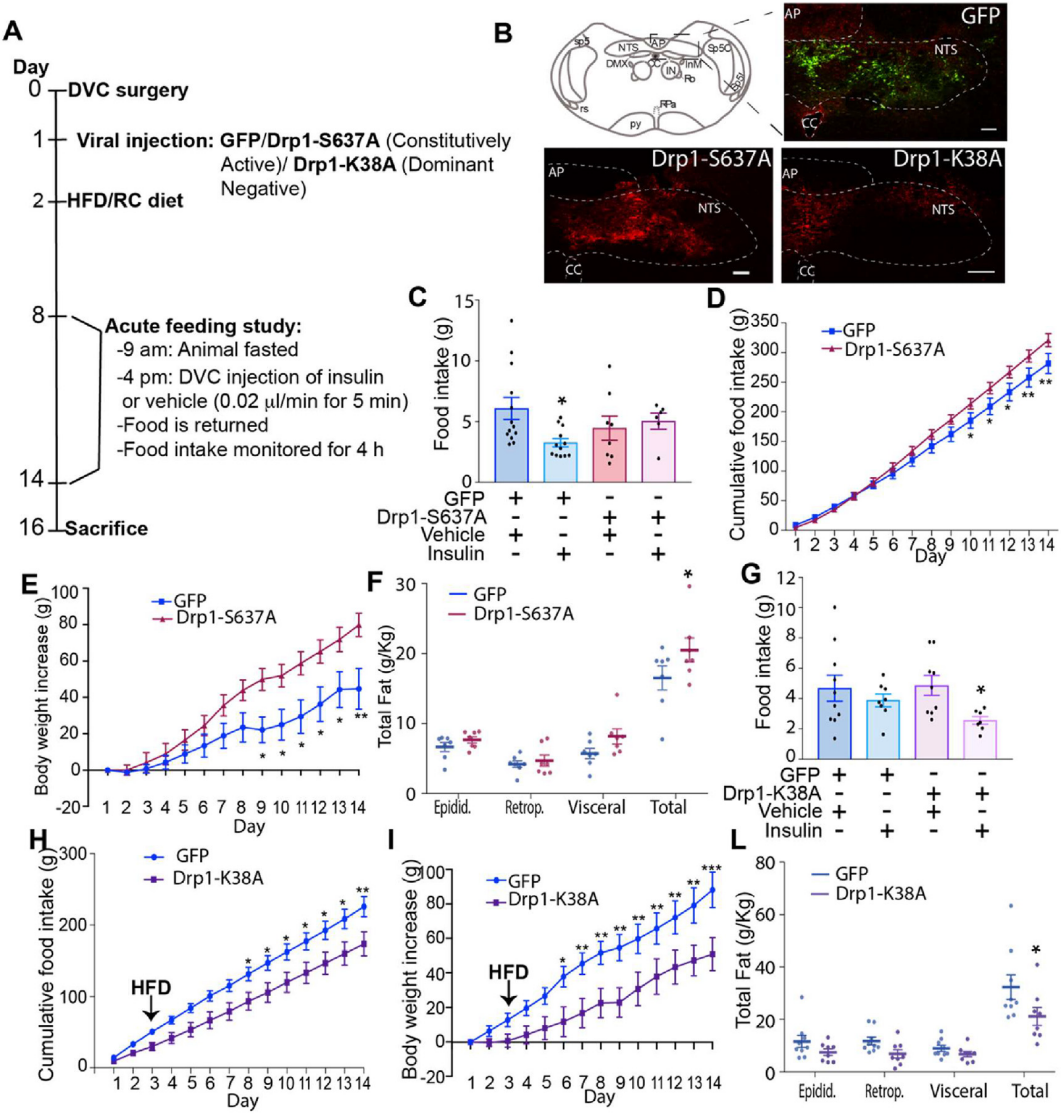
**Changes in mitochondrial fission in the DVC affect insulin sensitivity, food intake, body weight, and fat deposition.** (**A**) The experimental design including the feeding study procedure. (**B**) Representative confocal image of DVC areas expressing FLAG-tagged mutants of Drp1 (constitutively active Drp1-S637A and dominant negative Drp1-K38A) or GFP, in the NTS of the DVC. Scale bar = 100 μm. (**C and G**) Acute feeding study: animals fed RC (C) or an HFD (G) were fasted for 7 h and then infused bilaterally into the DVC with 0.2 μl of insulin or a vehicle over 5 min. Food was then returned, and food intake was observed every half hour for 4 h. Data are shown as mean ± SEM, with each single point highlighted. In C, data are representative of n = 13 for GFP vehicle, n = 12 for GFP insulin, n = 8 for Drp1-S637A vehicle, and n = 6 for Drp1-S637A insulin. In G, data are representative of n = 10 for GFP vehicle, n = 8 for GFP insulin, n = 9 for Drp1-S637A vehicle, and n = 7 for Drp1-S637A insulin. (**D and H**) Cumulative food intake from day 1. (**E and I**) Body weight increased from day 1. (**F and L**) White adipose tissue measurements: epididymal, retroperitoneal, and visceral fat collected on the day of sacrifice. Data are shown as mean ± SEM, with each single point highlighted. Data are representative of n = 7 rats for both GFP and Drp1-S637A in D to F and n = 9 rats for GFP and n = 8 rats for Drp1-K38A in H to L.∗p < 0.05, ∗∗p < 0.01, ∗∗∗p < 0.001.

The DVC senses insulin and decreases food intake; this effect is lost in HFD-fed, insulin-resistant rats. Increased mitochondrial fission was observed in the DVC of HFD-fed rats [28], and we attempted to determine whether HFD-dependent increased mitochondrial fission in the DVC affects the ability of insulin to lower food intake. In addition, we aimed to determine whether chronic alteration of mitochondrial dynamics in the DVC affects the feeding behaviour of rats. Using an adenoviral system, we expressed a CMV-driven constitutively active form of Drp1 (Drp1-S673A) in the NTS of the DVC, which increases mitochondrial fission [28]. A GFP-expressing adenovirus was used as control (Figure 1 A and B). Compared with GFP, expression of Drp1-S637A in HEK293AD cells decreased the aspect ratio (AR; Fig. S1 A, B, and D), suggesting that rounded mitochondria are related to increases in fission. Branching of mitochondria was evaluated by measuring the form factor (FF); we observed a slight but nonsignificant decrease in FF (Fig. S1E). In addition, Drp1-S637A colocalised with the mitochondria, as we expected, from an active isoform of Drp1 [28] (Fig. S1F).

Following viral injection, we monitored food intake and body weight for 2 weeks (Figure 1A). To determine whether changes in mitochondrial fission affect the ability of DVC insulin to decrease food intake, in weeks 1 and 2, we performed an acute feeding study: insulin was injected in the DVC (targeting the NTS) and food intake was monitored for 4 h (Figure 1A). Rats expressing Drp1-S637A were insulin resistant, hyperphagic, had increased body weight, and accumulated more fat than the GFP-expressing control rats did (Figure 1C–F). A single injection of insulin in the DVC was sufficient to decrease food intake in RC-fed rats within 4 h. Expression of the active form of Drp1 in the DVC induced insulin resistance and impaired the insulin-dependent decrease in food intake in RC-fed rats (Figure 1C). In addition, chronic activation of Drp1 in the DVC was sufficient to induce hyperphagia (Figure 1D), increase body weight (Figure 1E), and increase total white adipose tissue accumulation (sum of retroperitoneal, epididymal and visceral fat; Figure 1F). Increased mitochondrial fission in the DVC can induce ER stress [28]; we confirmed in our experimental conditions that expression of Drp1-S637A to increase mitochondrial fission increased phosphorylated PERK (a marker of ER stress; Fig. S2 A)

These data show that increasing Drp1-dependent mitochondrial fission by constitutive activation of Drp1 in the DVC is sufficient to cause insulin resistance and increase food intake and body weight gain.

### 3.2 Inhibition of mitochondrial fission in the DVC protects from developing HFD-dependent insulin resistance and decreases body weight and food intake

Three days of HFD feeding are sufficient to cause insulin resistance in the DVC and prevent an insulin-dependent decrease in food intake. There is also an increase in mitochondrial fission after 3 days of HFD feeding [28]; therefore, we aimed to determine whether reducing mitochondrial fission could protect rats from losing insulin sensitivity and affect feeding behaviour. To this end, we used a dominant negative form of Drp1, Drp1-K38A [28], which when expressed in HEK293AD cells it increases AR and FF, suggesting elongated and branched mitochondria, typical of reduced fission events (Fig. S1 A, C, D, and E). Being an inactive form, Drp1-K38A did not co-localise with mitochondria [28] (Fig. S1G). Inhibition of mitochondrial fission in the DVC of HFD-fed rats (by expressing Drp1, Drp1-K38A, Figure 1B) was sufficient to maintain insulin sensitivity and decrease food intake, body weight, and fat deposition (Figure 1G to L). More specifically, a single injection of insulin in the DVC was unable to decrease food intake in HFD-fed rats, and expression of the catalytically inactive form of Drp1 in the DVC of HFD-fed was sufficient to maintain insulin sensitivity and decrease food intake (Figure 1G). In addition, chronic inhibition of Drp1 in the DVC (through injection of the catalytically inactive Drp1-K38A) led to a decrease in food intake and body weight (Figure 1H,I) and decreased total white adipose tissue accumulation (sum of retroperitoneal, epididymal and visceral fat; Figure 1L). We also observed a decrease in ER stress (p = 0.055) in the DVC of HFD-fed rats expressing Drp1-K38A (Fig. S2B).

Overall, these data show that HFD-induced changes in mitochondrial dynamics can affect insulin sensitivity in the DVC and feeding behaviour and that inhibition of Drp1-dependent mitochondrial fission is protective against these deleterious changes.

### 3.3 Drp1 activity regulates iNOS levels in DVC

**Figure 2:**
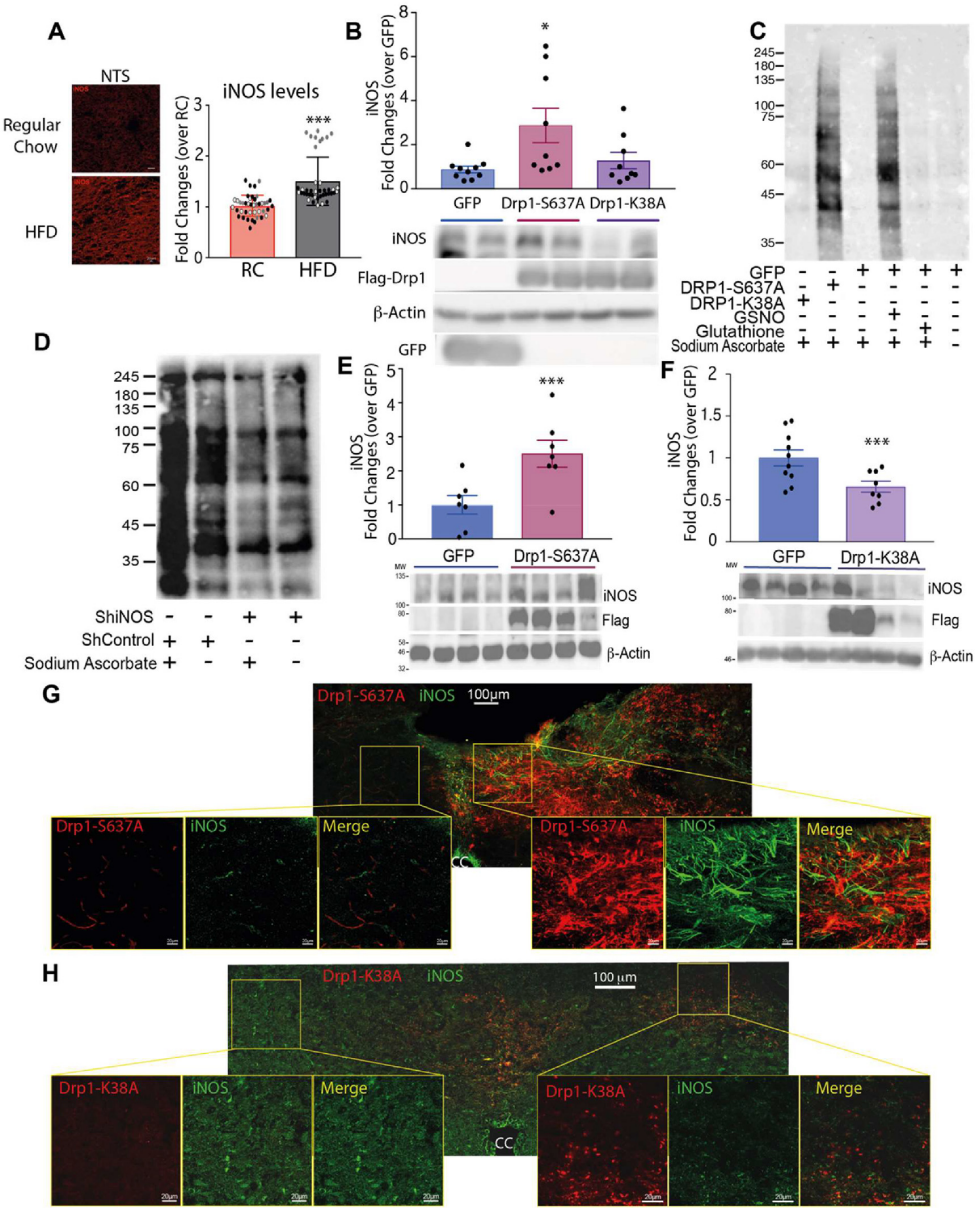
**Activation of Drp1 induces an increase in nitrosylated proteins and iNOS, and inhibition of Drp1 in HFD-fed rats decreases iNOS levels. (A)** Representative immunofluorescent (IF) images (see also Fig. S2) with quantification of iNOS levels in the NTS of rats fed with RC or HFD. Data are the average of 42 images taken from 3 pairs (RC vs HFD-fed) of rats; the single point is highlighted in the graph with a different colour, depending on the pair. Bar = 20 μm **(B)** iNOS levels in PC12 cells expressing GFP, Drp1-S637A, or Drp1-K38A. **(C)** Nitrosylation levels in PC12 cells expressing either GFP, Drp1-S637A, or Drp1-K38A. GFP-expressing cells were treated also with 200 mM of S-nitroglutathione or glutathione as a positive and negative control, respectively. Samples were reduced by using sodium ascorbate to enable specific labelling of nitrosylated proteins with iodoTMTzero. Sample without sodium ascorbate treatment showing non-specific staining **(D)** Nitrosylation levels of PC12 knocked down for iNOS compared with control PC12 cells (see also Fig. S4B). Sample without sodium ascorbate treatment showing non-specific staining **(E)** Western blot analysis of the changes in iNOS levels in RC-fed animals expressing GFP or Drp1-S637A in the DVC (same rats used in Figure 1 C–F). Data are shown as mean ± SEM, with each single point highlighted of n = 8 rats for both GFP and Drp1-S637A. **(F)** Western blot analysis of the changes in iNOS levels in HFD-fed animals expressing GFP or Drp1-K38A in the DVC (same rats used in Figure 1 G to L). Data are shown as mean ± SEM, with each single point highlighted of n = 8 for both GFP and Drp1-K38A. **(G**–**H)** IF representative images of iNOS levels in the NTS of RC-fed rats expressing Drp1-S637A (G) or HFD-fed rats expressing Drp1-K38A (H). ∗p < 0.05, ∗∗p < 0.01 ∗∗∗p < 0.001.

How changes in mitochondrial dynamics can affect insulin sensitivity remains unclear. Interestingly, we observed, both by IF and western blot, an increase in iNOS levels in the NTS of the DVC in HFD-fed rats compared with RC-fed rats (Figure 2A, Figs. S3 and S4A). Therefore, we investigated whether changes in mitochondrial dynamics affect iNOS levels. In the rat neuronal cell line PC12, expression of the active form of Drp1 (Drp1-S637A) led to an increase of iNOS levels (Figure 2B) when compared with the expression of the dominant negative form of Drp1 (Drp1-K38A) or GFP (Figure 2B). Changes in iNOS levels in the brain can cause an associated increase in nitric oxide (NO) levels. NO can act as a neurotransmitter or is used to nitrosylate cysteine (S-nitrosylation) or tyrosine (Nytril-tyrosine-N-Tyr) residues in a plethora of signalling molecules, changing their activity [30,[41], [42], [43]]. In the DVC of HFD-fed rats, increased iNOS levels were associated with higher tyrosine nitration (N-Tyr; Fig. S4A).

Next, we analysed the S-nitrosylation levels of proteins upon expression of Drp1-S637A, Drp1-K38A, and GFP in PC12 cells, and as predicted, cell lysates expressing Drp1-S637A to increase mitochondrial fission exhibited an increase in S-nitrosylated proteins when compared with cell lysates expressing Drp1-K38A or GFP (Figure 2C). Nitrosylation levels in cells expressing Drp1-S637A were comparable with those of the positive control (cells treated with 200 mM of S-nitroglutathione); no nitrosylation was observed in cells expressing GFP and Drp1-K38A or treated with glutathione (negative control). Nitrosylated protein levels were decreased in PC12 cells when iNOS was knocked down by using a lentiviral system expressing shRNA for iNOS (shiNOS), confirming that changes in iNOS levels can alter the nitrosylation pattern in PC12 cells (Figure 2D and Fig. S4B). Thus, the changes in nitrosylation levels could be a consequence of the higher iNOS expression levels in PC12 cells expressing Drp1-S367A. In summary, our results show that increasing mitochondrial fission is sufficient to increase iNOS levels and consequently increase S-nitrosylated proteins in PC12 neuronal cells.

Next, we investigated whether changes in mitochondrial dynamics affect iNOS levels in vivo. Expression of the active form of Drp1 in the DVC of RC-fed rats was sufficient to increase iNOS levels ∼2.5 times, and expression of the inactive form of Drp1 decreased iNOS levels ∼36% in the DVC of HFD-fed rats (Figure 2E,F). These data were also confirmed by IF; in RC-fed rats, NTS areas expressing Drp1-S637A presented higher iNOS protein levels than areas of the DVC where Drp1-S637A was not expressed did (Figure 2G). By contrast, in HFD-fed rats, the NTS areas expressing the inactive form of Drp1 presented lower iNOS protein levels than other DVC areas where Drp1-K38A was not expressed (Figure 2H). Altogether, our findings suggest that changes in iNOS levels caused by altered mitochondrial dynamics could be involved in the development of insulin resistance in the DVC.

### 3.4 iNOS knockdown in the DVC prevents HFD-dependent insulin resistance, hyperphagia, and body weight gain

**Figure 3:**
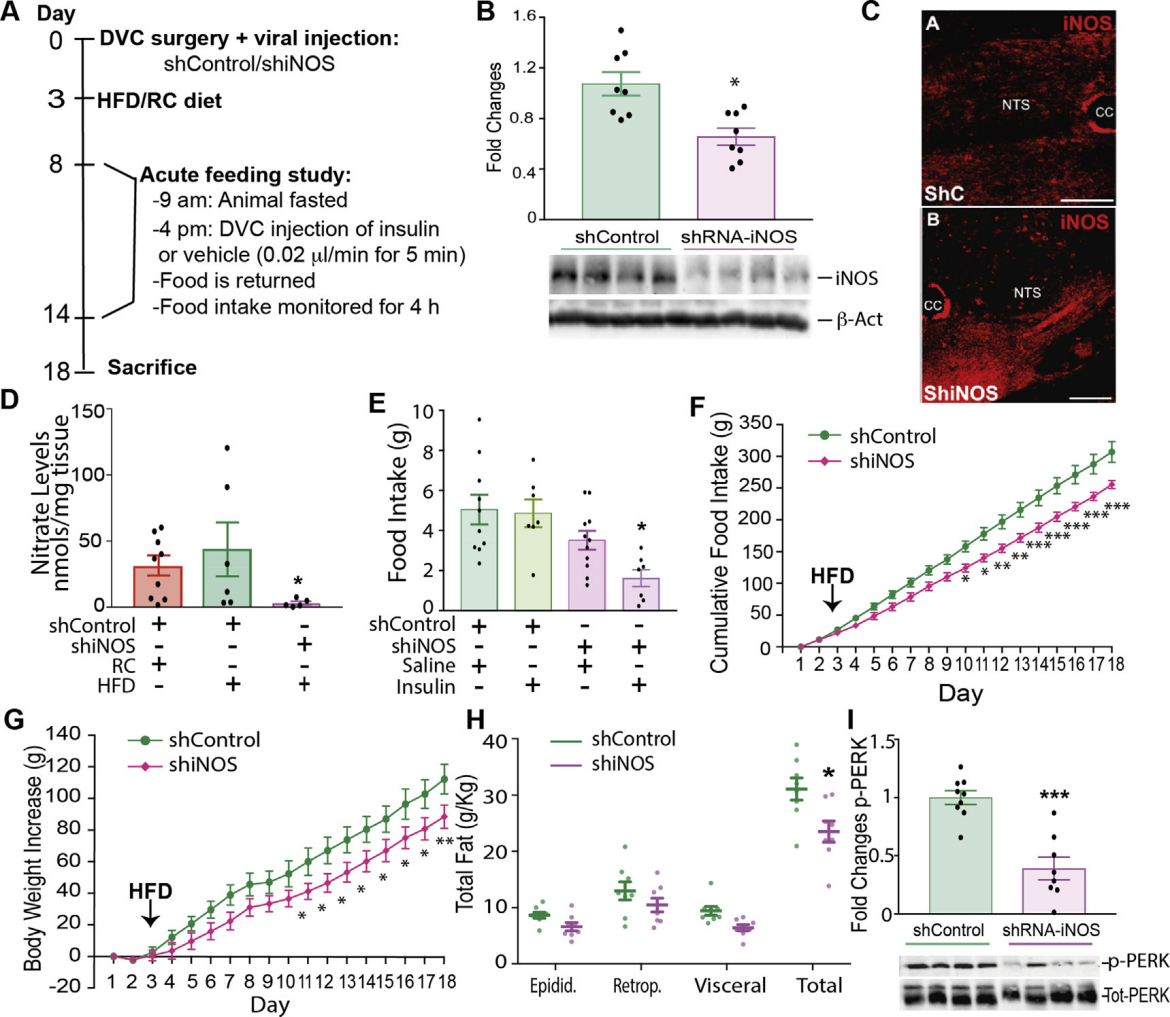
**Knockdown of iNOS in the DVC protects from developing HFD-dependent insulin resistance and decreases body weight and food intake.** (**A**) Experimental design and feeding study protocol. (**B**) Western blot analysis of the iNOS knockdown in the DVC. iNOS levels of n = 8 for shControl and n = 8 shiNOS are shown in the bar graph. A representative Western blot image is shown at the bottom. (**C**) Representative confocal image of iNOS labelling in animals expressing ShRNA for iNOS or the shControl in the NTS of the DVC. Bar = 20 μm (**D**) NO levels in the DVC of RC-fed rats compared with HFD-fed rats expressing the control virus and HFD-fed rats expressing shiNOS. Data are shown as mean ± SEM, with each single point highlighted of n = 9 RC rats and n = 6 HFD-fed rats expressing either shControl or shiNOS. (**E**) Acute feeding study: total food intake at 4 h, comparing animals treated with insulin or a vehicle in the DVC. Data are shown as mean ± SEM, with each single point highlighted of n = 10 rats for control vehicle, n = 7 for control insulin, n = 11 for shiNOS vehicle, n = 7 for shiNOS insulin. (**F**) Chronic cumulative food intake, from day 1 (see schematic in A). (**G**) Chronic data showing body weight increase from day 1. (**H**) White adipose tissue: epididymal, retroperitoneal, and visceral fat collected on the day of sacrifice. Data are shown as mean ± SEM, with each single point highlighted. Data from F to H are representative of n = 10 for shControl and n = 8 for shiNOS. (**I**) Western blot analysis of p-PERK levels in the DVC of animals expressing either ShiNOS or ShControl of n = 8 rats per group. A representative western blot image is shown at the bottom. ∗p < 0.05, ∗∗p < 0.01, ∗∗∗p < 0.001, ∗∗∗∗p < 0.0001.

Our data indicate that increasing mitochondrial fission in the DVC is sufficient to cause elevated iNOS levels. Studies have shown that increased iNOS levels can cause hypothalamic insulin resistance and obesity in mice [31]. We hypothesised that increased iNOS levels and a consequent increase in S-nitrosylation could be the link between mitochondrial fission and insulin resistance in the DVC. To investigate this possibility, we knocked down iNOS expression in the DVC by injecting a lentivirus expressing shiNOS (Figure 3A). Control rats were injected with a virus expressing scrambled shRNA (shControl). Using this approach, we observed that iNOS levels in the DVC were decreased by 50% (Figure 3B,C). To see whether changes in iNOS protein levels can affect the NO levels in the DVC, we collected DVC wedges from rats infected with either the shiNOS-expressing virus or the shControl-expressing virus and fed either with RC or HFD. NO is a volatile compound difficult to measure in tissues; however, a reliable estimation is possible by measuring NO metabolites such as nitrates [44]. We confirmed that knockdown of iNOS in the DVC led to a significant decrease in nitrate levels, suggesting that our approach was effective in decreasing both iNOS levels and activity (Figure 3D).

Next, we examined whether iNOS knockdown in the DVC could protect HFD-fed rats from developing insulin resistance. HFD-fed rats with decreased levels of iNOS in the DVC responded to a single injection of insulin in the DVC by decreasing food intake; HFD-fed rats expressing the control virus remained insulin resistant (Figure 3E). In addition, the chronic decrease of iNOS levels in HFD-fed rats (within 16 days from viral infection-Figure 3A), reduced food intake (Figure 3F), body weight gain (Figure 3G), and total white adipose tissue accumulation (sum of retroperitoneal, epididymal, and visceral fat; Figure 3H). Interestingly, knocking down iNOS in the DVC decreased ER stress, as indicated by lower levels of phosphorylated PERK (Figure 3I).

In summary, decreasing iNOS levels in the DVC is sufficient to prevent insulin resistance and reduce food intake, body weight gain, and fat deposition in HFD-fed rats.

### 3.5 Inhibition of Drp1 or knockdown of iNOS in the DVC restores insulin sensitivity in obese rats

**Figure 4:**
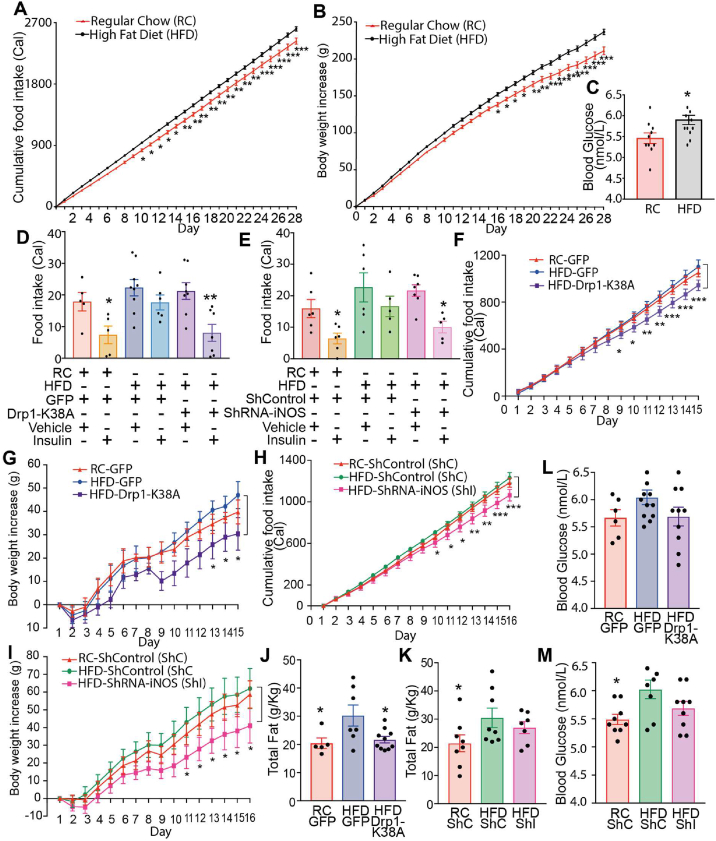
**Inhibiting mitochondrial fission and knocking down iNOS in obese rats successfully restored insulin sensitivity and decreased body weight and food intake.** Rats were fed for 28 days with HFD or control RC diet. On day 28 rats received DVC surgery. ShControl (ShC) and ShiNOS (ShI) virus were injected on surgery day; the GFP and Drp1-K38A viruses were injected on day 29. An acute feeding study was performed 8 and 14 days after surgery (Fig. S3). **(A)** Body weight increased over 4 weeks in HFD-fed compared with RC-fed animals **(B)** Cumulative food intake over the 4 weeks pre-surgery. Values are multiplied by calories of diet: 3.93 kcal/g RC, 5.51 kcal/g HFD. **(A and B)** Data are expressed as mean ± SEM, n = 10 RC, n = 24 HFD **(C)** Blood glucose pre-surgery. Bar charts represent mean ± SEM of individual rats, shown as single points. **(D and E)** Acute feeding study in GFP and Drp1-K38A-expressing animals (D) and ShC- and ShI-expressing animals (E). Graph shows the total food intake at 4 h. Bar charts represent mean ± SEM of individual rats shown as single points (n = 5 for RC GFP vehicle, n = 5 RC GFP insulin, n = 9 for HFD GFP vehicle, n = 6 for HFD GFP insulin, n = 8 for HFD Drp1-K38A vehicle, n = 6 for HFD Drp1-K38A insulin; n = 6 RC ShC insulin, n = 6 for HFD ShCl vehicle, n = 5 for HFD ShC insulin, n = 7 for HFD ShI vehicle, n = 5 for HFD ShI insulin). **(F and H)** Cumulative food intake starting from the day after surgery. **(G and I)** Body weight increase starting from the day after surgery **(G)**. **(J and K)** Total white adipose tissue (sum of epididymal, retroperitoneal, and visceral fat) collected on the day of sacrifice. Data are expressed as mean ± SEM, of n = 5 GFP RC, n = 6 GFP HFD, n = 10 Drp1-K38A HFD (F, G, and L) and of n = 8 ShC RC, n = 8 ShC HFD, n = 7 ShI HFD (H, I, K). **(L and M)** Average blood glucose over 14 days of the study, data is an average of sampled readings taken before feeding studies and day of sacrifice. Data are expressed as mean ± SEM, n = 6 GFP RC, n = 15 GFP HFD, n = 12 Drp1-K38A HFD and n = 9 for ShC RC, ShC HFD, ShI HFD. ∗p < 0.05, ∗∗p < 0.01, ∗∗∗p < 0.001.

Our data show that Drp1 inhibition or iNOS knockdown in the NTS of the DVC is sufficient to prevent the development of insulin resistance in a short- term HFD-fed model where an HFD is delivered after the viral injection. This finding suggests that our experimental approach may also be beneficial in an obese model. Therefore, we determined whether insulin sensitivity in an HFD-fed obese rat model could be restored by treating animals with a molecular inhibitor of Drp1 or by knocking down iNOS in the DVC. We used a well-established obese model: rats were fed an HFD for 4 weeks (28-days) [[36], [37], [38]]. Twenty-eight days of HFD feeding led to a sustained increase in calorie intake (Figure 4A) and a ∼10% increase in body weight when compared with RC-fed rats (Figure 4B). Blood glucose levels were also elevated in obese rats when compared with RC-fed rats (Figure 4C).

On day 28, we performed brain surgery and viral injection. Drp1-K38A or GFP injection was conducted the day after surgery (Fig. S5) and delivery of shiNOS or shControl was conducted on the day of the surgery (Fig. S5). We first investigated whether inhibition of mitochondrial fission or reduction of iNOS levels in the 28-day HFD-fed model could restore insulin sensitivity by performing a feeding study on days 9 and 14 after surgery (Fig. S5). As expected, infusion of insulin in the DVC of RC-fed control animals (expressing either GFP or shControl in the DVC) successfully decreased food intake, while our obese model was insulin resistant (Figure 4D,E). Notably, animals expressing the inactive form of Drp1 (Drp1-K38A) had a reduction in food intake in response to DVC insulin treatment, which was a response similar to that of RC control littermates (Figure 4D). Similarly, obese animals expressing the shiNOS vector to knockdown iNOS in the DVC presented with decreased food intake in response to DVC insulin treatment for 4 h, and their obese control littermates exhibited insulin resistance (Figure 4E). Overall, these data suggest that either inhibiting Drp1 or decreasing iNOS protein levels in the DVC is sufficient to restore insulin sensitivity in HFD-fed obese rats.

### 3.6 Chronic Drp1 inhibition or iNOS knockdown in DVC reverses obesity in rats

Next, we analysed the effect that chronic inhibition of Drp1 or iNOS knockdown in the DVC of obese rats has on food intake, body weight, blood glucose levels, and fat deposition. Although there was little difference in food intake and body weight between RC-fed and HFD-fed control rats expressing GFP, the rats expressing Drp1-K38A presented with a significant decrease in food intake, associated with a decrease in body weight gain (Figure 4F,G). We also observe a trend toward a decrease in blood glucose when compared with the HFD-fed rats (Figure 4L). Similarly, iNOS knockdown caused a decrease in body weight, food intake, and blood glucose levels (Figure 4H,G, and M). HFD-fed, obese rats also showed a significant increase in total adipose tissue deposition compared with that of RC-fed rats (Figure 4J,K). Drp1 inhibition in the DVC led to a decrease in total fat to the RC-fed level, whereas the iNOS knockdown led to a trend towards fat decrease, although this was not statistically significant (Figure 4J,K).

Collectively, our data suggest that inhibition of mitochondrial fission through Drp1 or iNOS knockdown in the DVC can restore insulin sensitivity; decrease body weight gain, food intake, and fat deposition in diet-induced obese rats; and moderately decrease in glycemia.

### 3.7 Inhibiting mitochondrial fission in DVC astrocytes of HFD-fed rats improves metabolic health

Our viral injection approach targets multiple cell populations in the NTS by expressing Drp1 under a CMV promoter. The cell population(s) in the DVC that are involved in insulin sensing and insulin resistance remain unknown. We analysed the cellular localisation of the GFP-expressing adenovirus and observed a high number of GFP-positive astrocytes (∼40%), followed by neurones (24%) and oligodendrocytes (∼36%; Figs. S6A–C). A similar pattern of expression was observed for Drp1-S637A- and Drp1-K38A-infected cells in the DVC (Fig. S7). Because astrocytes were highly targeted by our viral system, we first confirmed that alteration of mitochondrial dynamics in astrocytes affects both iNOS levels and phosphorylated PERK levels by using a rat brain astrocyte cell line: DI-TNC1 cells. Expression of Drp1-S637A in the astrocyte cell line increased iNOS levels and p-PERK, and expression of Drp1-K38A caused a significant decrease in iNOS levels and PERK phosphorylation (Figs. S8A and B). These data suggested that changes in mitochondrial dynamics can affect similar pathways in both neurones and astrocytes.

Astrocytes are the most abundant cell types in the brain and regulate multiple aspects of neuronal functions including synaptic plasticity, survival, metabolism and neurotransmission [45]. Interestingly, astrocytes also synthesise and release NO, used for communication with the surrounding neurones [41]. In addition, chemogenetic activation or inhibition of astrocytes in the DVC was reported to affect feeding behaviour [46].

**Figure 5:**
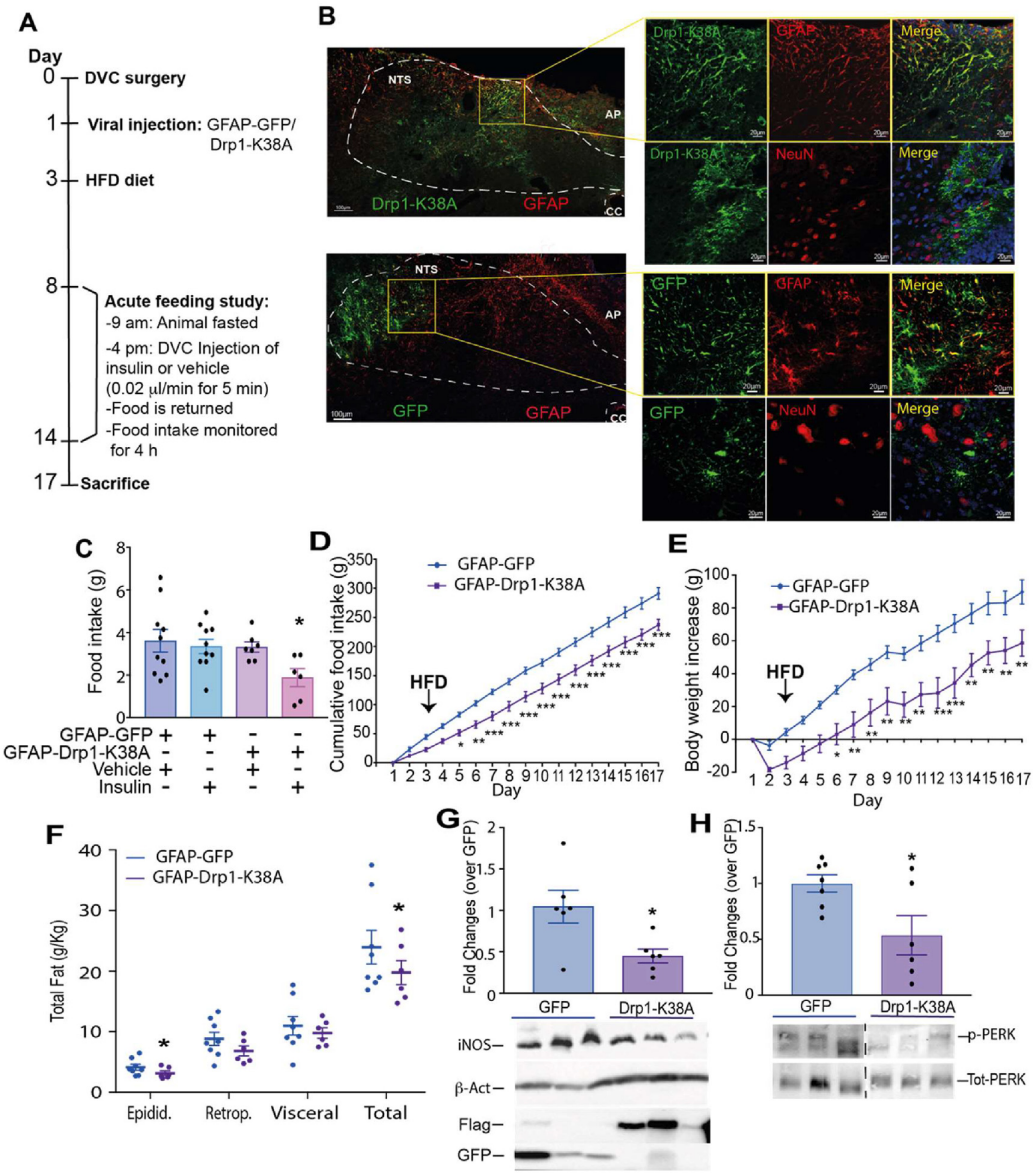
**Inhibition of mitochondrial fission in astrocytes of the DVC prevents the development of insulin resistance and decreases food intake, body weight gain, and fat deposition in HFD-fed rats.** (**A**) The experimental design, including the feeding study procedure. (**B**) Representative confocal image of DVC areas expressing FLAG-tagged Drp1-K38A or GFP under the GFAP promoter in the NTS of the DVC. Astrocytes are labelled with anti-GFAP antibody; neurones are labelled with anti-NeuN antibody. Bar = 100 μm for the full image and 20 μm for the magnified tiles (**C**) Acute feeding study: animals fed with an HFD were fasted for 6 h and then infused bilaterally into the DVC with a total 0.2 μl of insulin or a vehicle over 5 min. Food was then returned and food intake was observed every half hour for 4 h. Data are shown as mean ± SEM, with each single point highlighted. Data are representative of n = 10 for GFP vehicle, n = 10 for GFP insulin, n = 7 for Drp1-K38A vehicle, and n = 6 for Drp1-K38A insulin (**D**) Cumulative food intake from day of 1 (A). (**E**) Body weight increase from day 1. (**F**) White adipose tissue measurements: epididymal, retroperitoneal, and visceral fat collected on the day of sacrifice. Data are shown as mean ± SEM, with each single point highlighted. Data in D–F are representative of n = 7 rats for both GFP and Drp1-K38A. (**G, H**) Western blot analysis of changes in iNOS and phosphorylated PERK (p-PERK) levels in HFD-fed animals expressing GFP or Drp1-K38A in the astrocytes of the DVC. Representative western blot images of iNOS, β-actin, Flag, GFP, p-PERK, and Tot-PERK are also shown. Dotted line shows where the gel was cut. Data are shown as mean ± SEM, with each single point highlighted for n = 6 animals expressing GFP and n = 6 animals expressing Drp1-K38A. ∗p < 0.05, ∗∗p < 0.01, ∗∗∗p < 0.001.

These observations led to our hypothesis that altering mitochondrial dynamics in astrocytes may be sufficient to recapitulate the phenotypic changes we observed when targeting all cell types of the DVC. We tested this hypothesis by using a targeted adenoviral expression system under the control of the Glial Fibrillary Acidic Protein (GFAP) promoter [47]. Depending on the brain area, GFAP-expressing astrocytes can account for up to 60% of the total astrocyte population [48]. The NTS has a higher level of GFAP-expressing astrocytes [49] than DMX or AP do. We therefore generated viruses that express the dominant negative form of Drp1 (GFAP-Drp1-K38A) and a GFP control (GFAP-GFP) under the GFAP promoter. On day 0, rodents underwent a stereotactic surgery: a bilateral cannula was inserted into the NTS of DVC. On day one, rodents were injected with either a control virus expressing GFAP-GFP or GFAP-Drp1-K38A; on day 3, these rodents were given HFD for 14 days (Figure 5A). To investigate whether we achieved a targeted expression in astrocytes, we performed IF and confirmed the presence of co-localisation between GFAP-GFP or GFAP-Drp1-K38A and GFAP, while there was no co-localisation with a neuronal marker (NeuN; Figure 5B).

The feeding study was performed on days 8 and 14 (Figure 5A). Fasted rodents were injected with insulin or a vehicle bilaterally into the DVC, and food intake was measured every 30 min for 4 h (Figure 5A). Rodents expressing GFAP-Drp1-K38A had a 37% decrease in food intake in response to insulin compared with GFAP–GFP-expressing rodents that were insulin resistant (Figure 5C). Chronic inhibition of Drp1 in the astrocytes of the DVC decreased food intake and body weight (Figure 5D,E). Fat deposition was also decreased in rats expressing Drp1-K38A in astrocytes when compared with GFP-expressing rats (Figure 5F). These data show that inhibition of Drp1 in astrocytes of the DVC is sufficient to prevent HFD-dependent development of insulin resistance, hyperphagia, body weight gain, and fat accumulation. At the molecular level there was a reduction of iNOS levels in the DVC of HFD-fed rats expressing GFAP-Drp1-K38A when compared with GFAP–GFP-expressing rats (Figure 5G). ER stress levels were also significantly decreased in the DVC of rats expressing Drp1-K38A in astrocytes (Figure 5H).

Interestingly the effect on food intake and body weight was apparent from day 2, when rats were still fed the RC diet; these data suggested that also in an RC-fed rodent, inhibition of mitochondrial fission could affect feeding behaviour. To confirm our hypothesis, we performed a feeding study in RC-fed rats expressing either Drp1-K38A or GFP in astrocytes and confirmed that Drp1-K38A expression in astrocytes decreased both food intake (Fig. S8C) and body weight (Fig. S8D); no differences in fat deposition were observed (Fig. S8E).

In summary, the modulation of mitochondrial dynamics in astrocytes of the DVC is sufficient to regulate iNOS levels, insulin sensitivity, feeding behaviour, body weight, and fat deposition in rats.

## 4 Discussion

**Figure 6:**
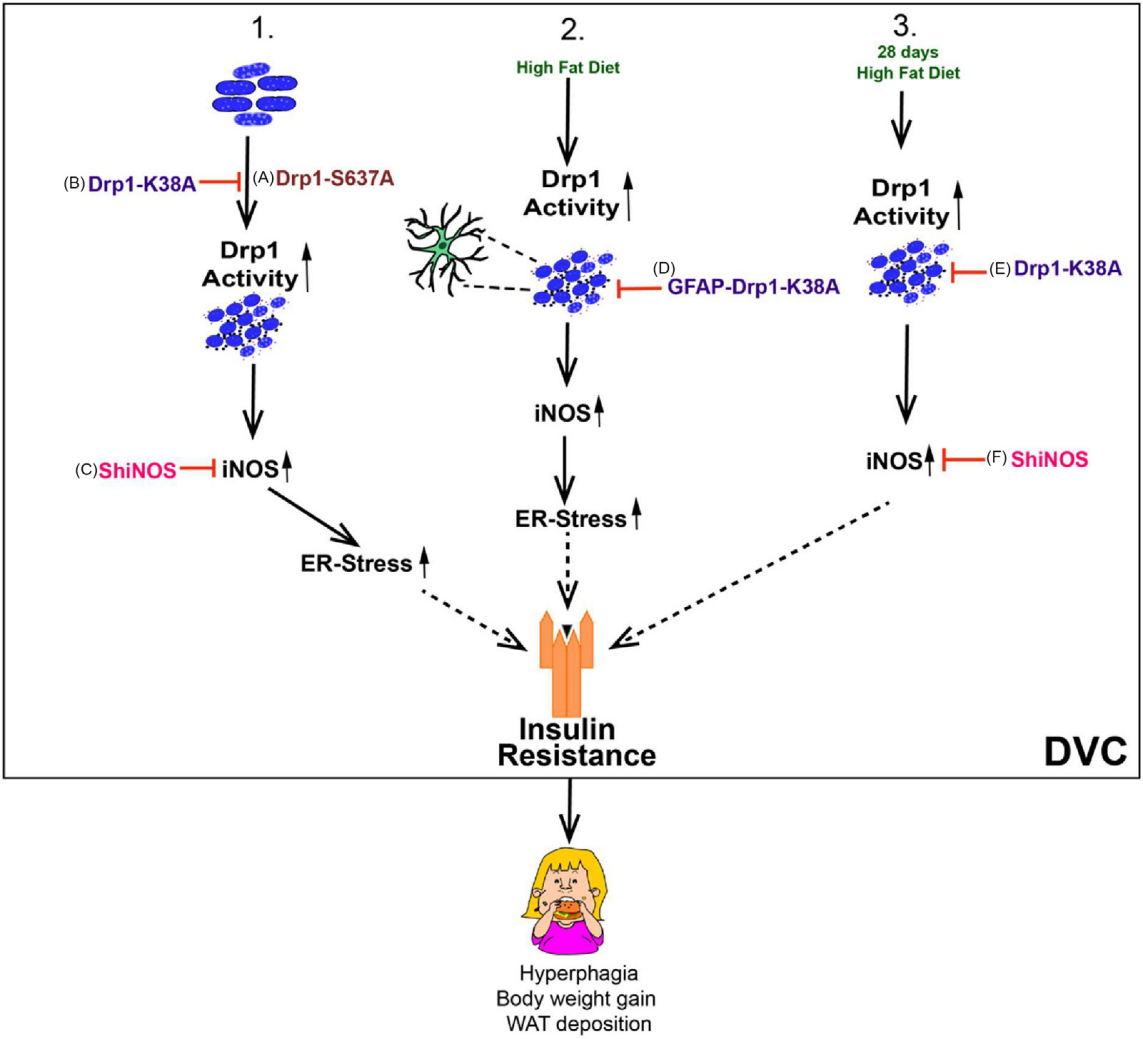
Summary of Drp1 regulation of insulin resistance and body weight. (1) Activation of Drp1 in the DVC causes insulin resistance, hyperphagia, and body weight gain (A), and inhibition of Drp1 (B) or decreasing iNOS levels (C) in the DVC is sufficient to protect from HFD-dependent insulin resistance, hyperphagia, and body weight gain. (2) Inhibition of mitochondrial fission in DVC astrocytes by expressing Drp1-K38A (D) protects HFD-fed rats from developing insulin resistance and lowers food intake, body weight, and fat deposition. (3) Inhibition of mitochondria fission (E) or knockdown of iNOS (F) can restore insulin sensitivity and decrease body weight gain and fat deposition in a 28-day HFD-fed obese model. Dotted lines = potential pathway.

We have shown that increasing DVC mitochondrial fission triggers insulin resistance, causes hyperphagia, and increases body weight gain and fat deposition (Figure 6A). Conversely, inhibiting DVC mitochondrial fission protects rats from developing HFD-dependent insulin resistance, hyperphagia, and body weight gain (Figure 6B). Using a 28-day HFD-fed obese model, we also demonstrated that inhibition of mitochondrial fission restores insulin sensitivity and decreases body weight and fat deposition after long-term HFD feeding (Figure 6E). Furthermore, we provided evidence that increased DVC mitochondrial fission leads to higher ER stress and iNOS levels. Knocking down iNOS in the DVC prevents insulin resistance in HFD-fed rats and reduces hyperphagia, body weight gain, and ER stress (Figure 6C, F). Finally, we showed that inhibiting mitochondrial fission in astrocytes is sufficient to protect HFD-fed rats from developing insulin resistance and results in lower food intake, lower body weight, and fat deposition (Figure 6D).

Our findings are consistent with those of studies that have demonstrated that alterations to mitochondrial dynamics in both anorexigenic (POMC) and orexigenic (AgRP) neuronal subtypes within the hypothalamus can affect feeding behaviours and glucose metabolism. Deletion of Drp1 in POMC neurons improves glucose metabolism [25]. Additionally, loss of Mfn-1 or Mfn-2 in POMC neurones leads to impaired glucose sensing and insulin release, defective insulin sensing, and an increase in reactive oxygen species (ROS) and ER stress [50,51]. By contrast, inhibition of Mfn-1 and 2 in AgRP neurons in the hypothalamus prevented diet-induced obesity in rodents fed an HFD [52].

Our data showed for the first time that activation of mitochondrial fission in the DVC can induce hyperphagia in RC-fed rodents. Furthermore, the inhibition of Drp1-dependent mitochondrial fission in the DVC of HFD-fed rodents prevented hyperphagia and body weight gain and restored insulin sensitivity in a diet-induced obesity model. Mitochondrial dynamics governed by Drp1 also regulates energy metabolism in other tissues, for instance, in HFD-fed and obese rodents, increased mitochondrial fragmentation in muscle cause insulin resistance and metabolic imbalance [[53], [54], [55]]. Thus, the mechanistic insights revealed here, are likely to be relevant to other organs and tissues and may represent a unifying model by which mitochondria regulate glucose metabolism, insulin signalling, and body weight.

We showed that Drp1 activation in the DVC leads to an increase in the total amount of WAT deposition in RC-fed rodents, and Drp1 inhibition in HFD-fed rodents resulted in lower WAT deposits. The increase in WAT levels caused by increased mitochondrial fission in the DVC could increase ROS and free fatty acids (FFA) levels, potentially exacerbating the insulin resistance by also affecting peripheral organ insulin sensitivity. In fact, an increase in FFA enhances the oxidation of adipose tissue, leading to an accumulation of lipids and mitochondrial dysfunction; this can increase oxidative stress ROS and inflammation in tissues, which are associated with insulin resistance and body weight gain [21].

Consistent with HFD increasing iNOS levels in the DVC [28], we demonstrated that mitochondrial fission increases iNOS levels in the DVC and that a reduction of iNOS levels can protect against the development of HFD-dependent insulin resistance and restore insulin sensitivity in an obese model. iNOS is a marker of inflammation [56,57], where HFD-fed rodents exhibit higher levels of iNOS in muscle leading to insulin resistance [58]. In addition, hypothalamic infusion of NO, to mimic high iNOS activity triggered insulin resistance and increased food intake [31]. Interestingly, mRNA levels of iNOS were increased in LPS-treated BV2 microglial cells; however, this phenotype was reversed by treatment with a Drp1 inhibitor, MDIVI-1 [59]. Altogether, these data indicate that iNOS levels are affected by changes in mitochondrial dynamics and that decreasing iNOS levels can restore insulin sensitivity. Further research is necessary to prove that when mitochondrial fission is elevated (e.g. by activating Drp1) decreasing the iNOS level is sufficient to restore insulin sensitivity, or whether an alternative pathway could be involved.

How changes in iNOS levels trigger insulin resistance and reduce body weight and food intake remains partially understood. We observed that shRNA-mediated knockdown of iNOS significantly reduced NO levels in the DVC of HFD-fed rats. In addition, *in vitro*, we could demonstrate that activation of Drp1 can trigger an increase in iNOS levels and nitrosylated proteins (Figure 2B,C). This is in agreement with studies of other tissues that have shown that the S-nitrosylation of insulin signalling-associated molecules in muscle (e.g., IRS-1 and AKT) or key ER stress-associated molecules (e.g., unfolded protein response-UPR-regulator, IRE1α) in the liver can trigger insulin resistance and obesity [29,58,60,61]. A marked increase in iNOS levels was also reported in mouse modes of diabetes, and iNOS knockout in HFD-fed mice led to improved glucose tolerance and insulin sensitivity in skeletal muscle [58]. Interestingly, infusion of NO or S-nitrosoglutathione into the hypothalamus resulted in insulin resistance and increased food intake [31]. Drp1 can also be S-nitrosylated under high oxidative stress, which can increase the rate of mitochondrial fission and change the energy balance [62]. Therefore, a potential molecular mechanism that triggers insulin resistance in the DVC could be associated with increased iNOS levels and increased nitrosylation of key molecules involved in the transduction of insulin signalling. Chronic reduction of iNOS levels in the DVC could prevent hyperphagia and body weight gain by mitigating the HFD-dependent nitrosative stress and S-nitrosylation of key players in the insulin signalling pathway.

Targeting mitochondrial dynamics in the brain could benefit obese subjects. Animal models of obesity exhibited altered mitochondrial dynamics, for example, Zucker rodents displayed decreased mitochondrial functionality, which correlated with a decrease in ATP production and an increase in Drp1 activity [63]. Increased calorie consumption also impaired mitochondrial function in the brain, leading to increased ROS production and UPR activation [64,65]. We showed that inhibiting Drp1 in the DVC of 28-day HFD-fed obese rats is sufficient to restore insulin sensitivity and decrease food intake and body weight. Notably, recovery after surgery was faster for RC-fed rats than HFD-fed rats; thus, in the first week post-surgery, difference in body weight between RC and HFD-fed control rats was minimal (Figure 4G,I). We also observed that decreasing iNOS levels in the DVC benefits obese rats. Notably, a whole-body iNOS knockout model presented with improved glucose homeostasis and increased sensitivity to insulin than their obese control counterparts [58]. Indeed, the treatment of the hypothalamus with an iNOS inhibitor, L-NIL, by ICV injection, inhibited HFD-induced astrogliosis, suggesting that iNOS activation may induce hypothalamic inflammation [66]. Therefore, our study strengthens the notion that iNOS may be a satisfactory target to ameliorate obese phenotypes.

A major question is which cell types in the DVC are responsible for insulin sensing and resistance. Using a specific targeting of cell types, we demonstrated that inhibition of mitochondrial fission in GFAP-expressing astrocytes of the DVC is sufficient to reduce food intake and body weight gain in HFD-fed and RC-fed rodents. Astrocytes provide metabolic and structural support to neurones and play an active role in neurotransmission. Changes in astrocyte number, density, and activity have been associated with HFD feeding, insulin resistance, and obesity [67]. Interestingly, astrocytes in the NTS of the DVC responded to acute nutritional overload by increasing their network complexity to integrate peripheral satiety signals to decrease food intake [46].

Therefore, we could hypothesise that there is a potential neuronal–glial cross talk in which astrocytes sense changes in nutritional levels and affect how surrounding neurones control energy homeostasis, potentially *via* NO release or altering the activity of key signalling molecules *via* nitrosylation.

Interestingly, we have also shown that inhibition of mitochondrial fission in GFAP-expressing astrocytes of the DVC has an important protective effect on the development of HFD-dependent insulin resistance. In the hypothalamus, ablation of insulin receptors in astrocytes affected glial morphology and mitochondrial function and reduced the activation of POMC neurones [45]. Whether changes in mitochondrial fission in astrocytes of the DVC affect how astrocytes sense insulin or how neurones respond to changes in insulin levels is an important question that warrants further investigation.

The DVC is an underappreciated area of the brain that integrates peripheral cues from the metabolic status of an individual and relays to the forebrain, to control and maintain energy balance. Here, we provide insights into a complex molecular mechanism that triggers insulin resistance in the DVC, to cause metabolic imbalance and highlight how the astrocytes, as energy sensors in the brain, play a major role in this process.

Our work uncovers new molecular and cellular targets that can be exploited to develop new approaches focusing on the brain to counteract the deleterious effects of obesity and diabetes.

## Author contributions

**B.P.** conducted and designed experiments, performed data analyses, and wrote the first manuscript draft. **L.N**. assisted with in vivo experiments, performed all histochemistry, and participated in the data analysis. **J.C.G.** assisted with the experiments. **J.D.** contributed to the experimental design, discussion of results, and critiqued the manuscript. **B.M.F.** conceived and supervised the project, designed the experiments (and conducted some), and wrote the manuscript.

## Appendix A Supplementary data

Multimedia component 1 **Figure S1**: Expression of Drp1-S637A or Drp1-K38A in HEK239AD cells alters mitochondrial dynamics. (**A-C)** Representative confocal images showing the expression of GFP (**A**), Drp1-S637A-FLAG (**B**), or Drp1-K38A-FLAG (**C**) (green) in HEK293AD cells treated with MitoTracker red CMXRos (red). Nuclei are stained with DAPI (Blue). (**Ai-Bi**) Binary images of MitoTracker labelling generated in Fiji to determine mitochondrial aspect ratio (AR) and form factor (FF) in GFP- (**Ai**), Drp1-S637A-FLAG- (**Bi**) and Drp1-K38 A-FLAG- **(Ci**) positive cells. Scale bars represent 10 μm. (**D**) Aspect ratio of mitochondria. (**E**) Form factor of mitochondria. Data are expressed as mean ± SEM, *n* = 106 cells for each condition, ∗∗∗∗p < 0.0001. **(F**–**G)** Co-localisation between Drp1 mutants and MitoTracker. Representative confocal images showing MitoTracker staining **(F & G),** Drp1-S637A-FLAG (**Fi**), Drp1-K38-FLAG (**Gi**) and Merge between MitoTracker and FLAG (**Fii & Gii**). Arrows represent co-localisation. Scale bars represent 10 μm. **Figure S2**: Phosphorylated PERK levels in the DVC of rats expressing either Drp1-S637A or Drp1-K38A: Western blot analysis of the changes in p-PERK levels in RC-fed animals expressing GFP or Drp1-S637A in the DVC (same rats used in Figure 1 C–F). Data are shown as mean ± SEM, with each single point highlighted of n = 6 rats for GFP and n = 5 rats for Drp1-S637A. **(F)** Western blot analysis of the changes in iNOS levels in HFD-fed animals expressing GFP or Drp1-K38A in the DVC (same rats used in Figure 1 G–L). Data are shown as mean ± SEM, with each single point highlighted of n = 8 for both GFP and Drp1-K38A. **Figure S3**: HFD increases iNOS levels in the DVC. Representative images of iNOS staining in the DVC of RC (**A**) and HFD-fed (**B**) rats. Image shows a large tile that includes a central canal and NTS of the DVC. A magnified area is also shown. **Figure S4**: HFD feeding increases iNOS and tyrosine nitration levels in the DVC and control blots for Figure 2 D. A. Western blot analysis of the changes in iNOS or Nitryl Tyrosine (N-Tyr) levels in the DVC of HFD-fed rats compared with RC-fed rats. Representative western blot images of iNOS, N-Tyr, and β-actin are also shown. **B.** PC12 cells infected with a lentivirus expressing shRNA for iNOS or Control scramble virus were lysed and iNOS knockdown levels are quantified by western blotting. In the figure the quantification with a representative western blot is shown. All data are expressed as mean ± SEM *n* = 3 for shControl and shiNOS-expressing cells. [∗p < 0.05, ∗∗p < 0.01]. **Figure S5**: Experimental design for the 28d HFD-fed obese model. Rats were fed for 28 days with an HFD or control RC diet. On day 28, rats received DVC surgery**.** shControl and shiNOS virus were injected on surgery day; the GFP and Drp1-K38A viruses were injected on day 29. An acute feeding study was performed on days 8 and 14 after surgery. **Figure S6**: GFP expression in neural cell types. (A-Aii) Representative confocal images illustrating labelling of GFP expression in (A), NeuN (Ai), and dual labelling (Aii) in the DVC. **(B-Bii)** Representative confocal images illustrating labelling of GFP expression in (B), GFAP (Bi), and dual labelling (Bii) in the DVC. **(C-Cii)** Representative confocal images illustrating labelling of GFP expression in (C), PanQKi (Ci), and dual labelling (Cii) in the DVC. **(D)** Quantification of the co-localised cells. Closed arrows denote co-localised cells. Images and quantification represent the average of n = 3 for NeuN and PanQKi, and n = 4 for GFAP animals. Three images were quantified per animal). **Figure S7**: Expression of the constitutively active form of Drp1, Drp1-S637A and the dominant negative form of Drp1, Drp1-K38A in neuronal cells. (A-Aii) Representative confocal images illustrating the expression Drp1-S637A (A), NeuN (Ai), and dual labelling (Aii) in the DVC. Blue in Aii represents Nuclei stained with Dapi. **(B-Bii)** Representative maximal intensity confocal images illustrating the expression of Drp1-K38A (B), NeuN (Bi), and dual labelling plus Dapi in blue (Bii) in the DVC. **(C-Cii)** Representative confocal images illustrating the expression of Drp1-S637A (C), GFAP (Ci), and dual labelling plus Dapi in blue (Cii) in the DVC. **(D-Dii)** Representative confocal images illustrating the expression of Drp1-K38A (D), GFAP (Di), and dual labelling plus Dapi in blue (Dii) in the DVC. **(E-Eii)** Representative confocal images illustrating the expression of Drp1-S637A (E), Iba1 (Ei), and dual labelling plus Dapi in blue (Eii) in the DVC. **(F-Fii)** Representative confocal images illustrating the expression Drp1-K38A (F), Iba1 (Fi), and dual labelling plus Dapi in blue (Fii) in the DVC. Open arrows denote non-colocalised cells. Closed arrows denote colocalised cells. **Figure S8**: Inhibition of mitochondrial fission in GFAP-expressing astrocytes decreases iNOS and p-PERK levels in HFD-fed rats; inhibition of mitochondria fission in astrocytes of the DVC decreases food intake, and body weight in regular chow-fed rats. (**A-B**) Western blot analysis of iNOS (A) and p-PERK (B) levels in astrocytes cell expressing GFP, Drp1-S637A, and Drp1-K38A. Data are the average of n = 7 samples for each condition. Representative western blot images show GFP, Flag-tagged Drp1 mutants, β-actin, iNOS, and p-PERK expression. **(C to E)** A double cannula was inserted into the NTS of the DVC in RC-fed rats on day 0. On day 1, rats were injected with an adenovirus expressing either GFAP-GFP or GFAP-Drp1-K38 in the NTS of the DVC. Food intake and body weight were measured daily for 17 days. (**C**) Cumulative food intake from day 1. (**D**) Body weight increase from day 1. (**E**) White adipose tissue measurements- epididymal, retroperitoneal, and visceral fat collected on the day of sacrifice. Data are shown min ±SEM, with each single point highlighted. Data are representative of n = 8 rats for both GFP and Drp1-K38A. ∗p < 0.05, ∗∗p < 0.01. Multimedia component 1
