# Supplementary material for: Inhibition of mitochondrial fission and iNOS in the dorsal vagal complex protects from overeating and weight gain
Source: Mol Metab. 2020 Nov 20;43:101123. doi: 10.1016/j.molmet.2020.101123 (PMC7753200; doi:10.1016/j.molmet.2020.101123)
Supplement: Multimedia component 1 — Figure S1: Expression of Drp1-S637A or Drp1-K38A in HEK239AD cells alters mitochondrial dynamics. (A-C) Representative confocal images showing the expression of GFP (A), Drp1-S637A-FLAG (B), or Drp1-K38A-FLAG (C) (green) in HEK293AD cells treated with MitoTracker red CMXRos (red). Nuclei are stained with DAPI (Blue). (Ai-Bi) Binary images of MitoTracker labelling generated in Fiji to determine mitochondrial aspect ratio (AR) and form factor (FF) in GFP- (Ai), Drp1-S637A-FLAG- (Bi) and Drp1-K38 A-FLAG- (Ci) positive cells. Scale bars represent 10 μm. (D) Aspect ratio of mitochondria. (E) Form factor of mitochondria. Data are expressed as mean ± SEM, n = 106 cells for each condition, ∗∗∗∗p < 0.0001. (F–G) Co-localisation between Drp1 mutants and MitoTracker. Representative confocal images showing MitoTracker staining (F & G), Drp1-S637A-FLAG (Fi), Drp1-K38-FLAG (Gi) and Merge between MitoTracker and FLAG (Fii & Gii). Arrows represent co-localisation. Scale bars represent 10 μm. Figure S2: Phosphorylated PERK levels in the DVC of rats expressing either Drp1-S637A or Drp1-K38A: Western blot analysis of the changes in p-PERK levels in RC-fed animals expressing GFP or Drp1-S637A in the DVC (same rats used in Figure 1 C–F). Data are shown as mean ± SEM, with each single point highlighted of n = 6 rats for GFP and n = 5 rats for Drp1-S637A. (F) Western blot analysis of the changes in iNOS levels in HFD-fed animals expressing GFP or Drp1-K38A in the DVC (same rats used in Figure 1 G–L). Data are shown as mean ± SEM, with each single point highlighted of n = 8 for both GFP and Drp1-K38A. Figure S3: HFD increases iNOS levels in the DVC. Representative images of iNOS staining in the DVC of RC (A) and HFD-fed (B) rats. Image shows a large tile that includes a central canal and NTS of the DVC. A magnified area is also shown. Figure S4: HFD feeding increases iNOS and tyrosine nitration levels in the DVC and control blots for Figure 2 D. A. Western blot analysis of the changes in i [file mmc1.docx]

**Supplementary Figures**

**
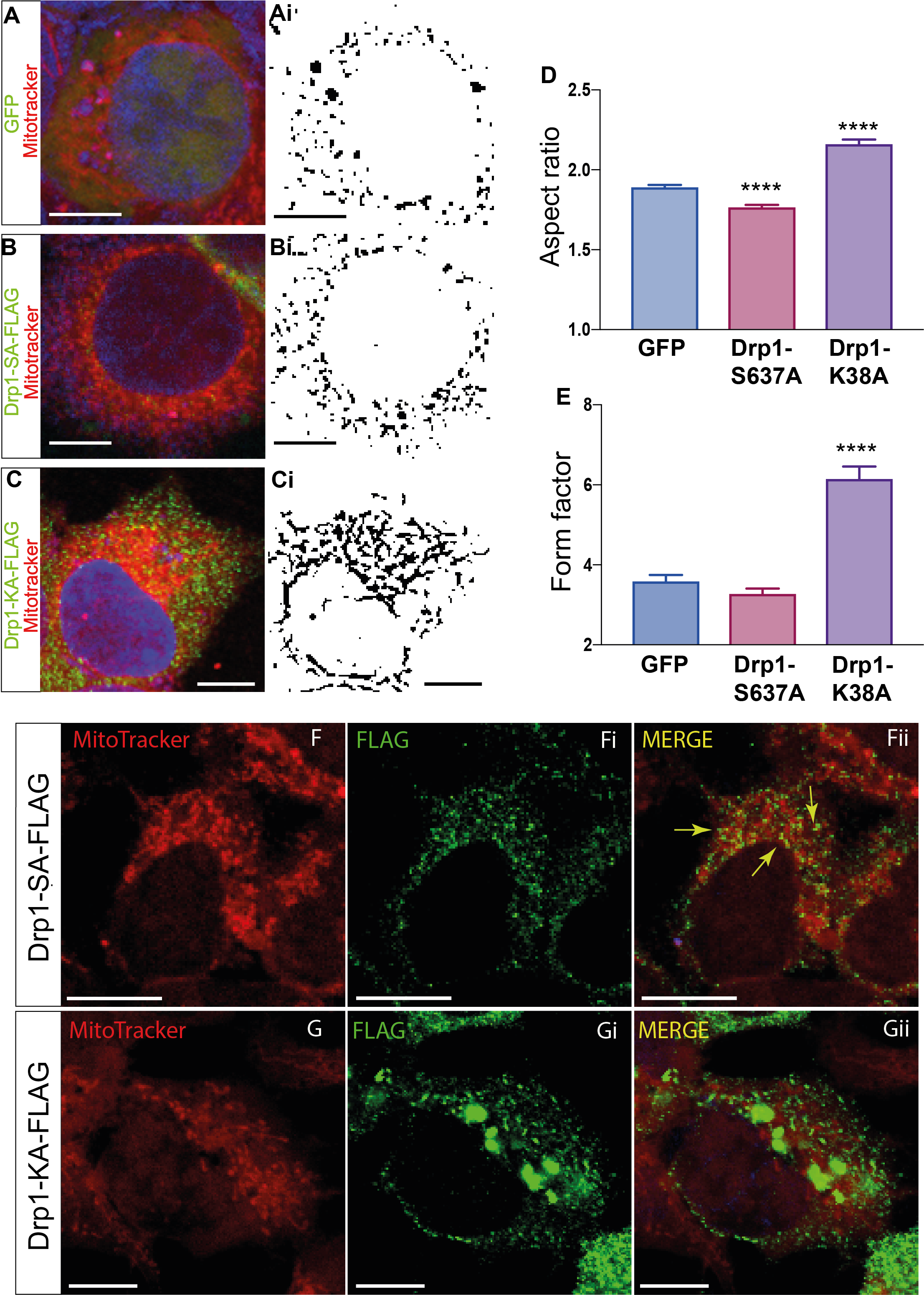
**

**Figure S1: Expression of Drp1-S637A or Drp1-K38A in HEK239AD cells alters mitochondrial dynamics.** (**A-C)** Representative confocal images showing the expression of GFP (**A**), Drp1-S637A-FLAG (**B**), or Drp1-K38A-FLAG (**C**) (green) in HEK293AD cells treated with MitoTracker red CMXRos (red). Nuclei are stained with DAPI (Blue). (**Ai-Bi**) Binary images of MitoTracker labelling generated in Fiji to determine mitochondrial aspect ratio (AR) and form factor (FF) in GFP- (**Ai**), Drp1-S637A-FLAG- (**Bi**) and Drp1-K38 A-FLAG- **(Ci**) positive cells.  Scale bars represent 10 μm. (**D**) Aspect ratio of mitochondria. (**E**) Form factor of mitochondria. Data are expressed as mean ± SEM, *n* = 106 cells for each condition, ****p<0.0001. **(F-G)** Co-localisation between Drp1 mutants and MitoTracker. Representative confocal images showing MitoTracker staining **(F & G),** Drp1-S637A-FLAG (**Fi**), Drp1-K38-FLAG (**Gi**) and Merge between MitoTracker and FLAG (**Fii & Gii**). Arrows represent co-localisation. Scale bars represent 10 μm.


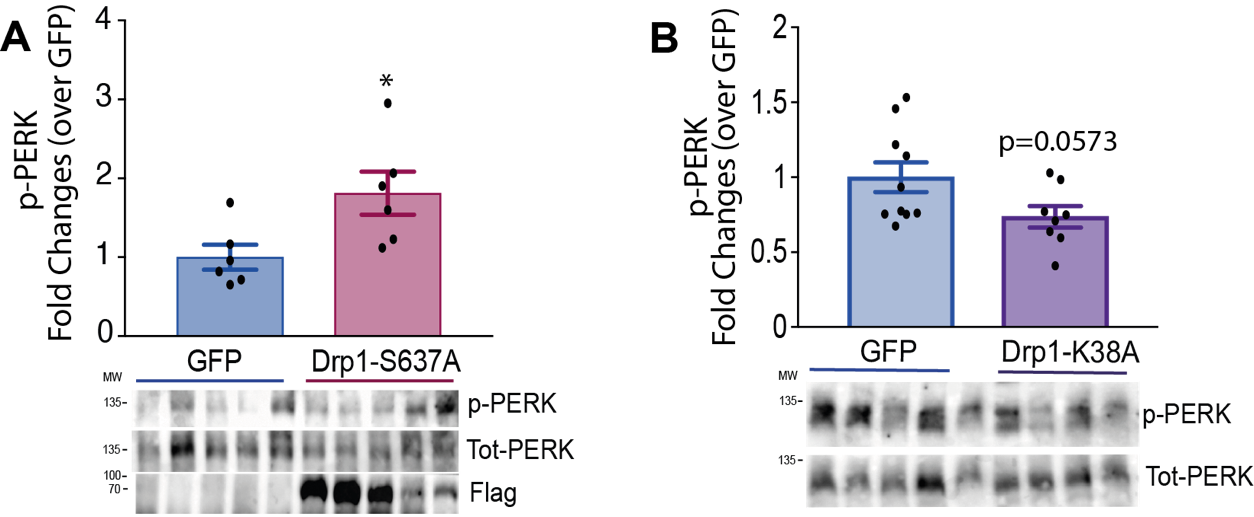


**Figure S2:** **Phosphorylated PERK levels in the DVC of rats expressing either Drp1-S637A or Drp1-K38A**: Western blot analysis of the changes in p-PERK levels in RC-fed animals expressing GFP or Drp1-S637A in the DVC (same rats used in Fig 1 C–F). Data are shown as mean ± SEM, with each single point highlighted of n=6 rats for GFP and n=5 rats for Drp1-S637A. **(F)** Western blot analysis of the changes in iNOS levels in HFD-fed animals expressing GFP or Drp1-K38A in the DVC (same rats used in Fig. 1 G–L). Data are shown as mean ± SEM, with each single point highlighted of n=8 for both GFP and Drp1-K38A.

**
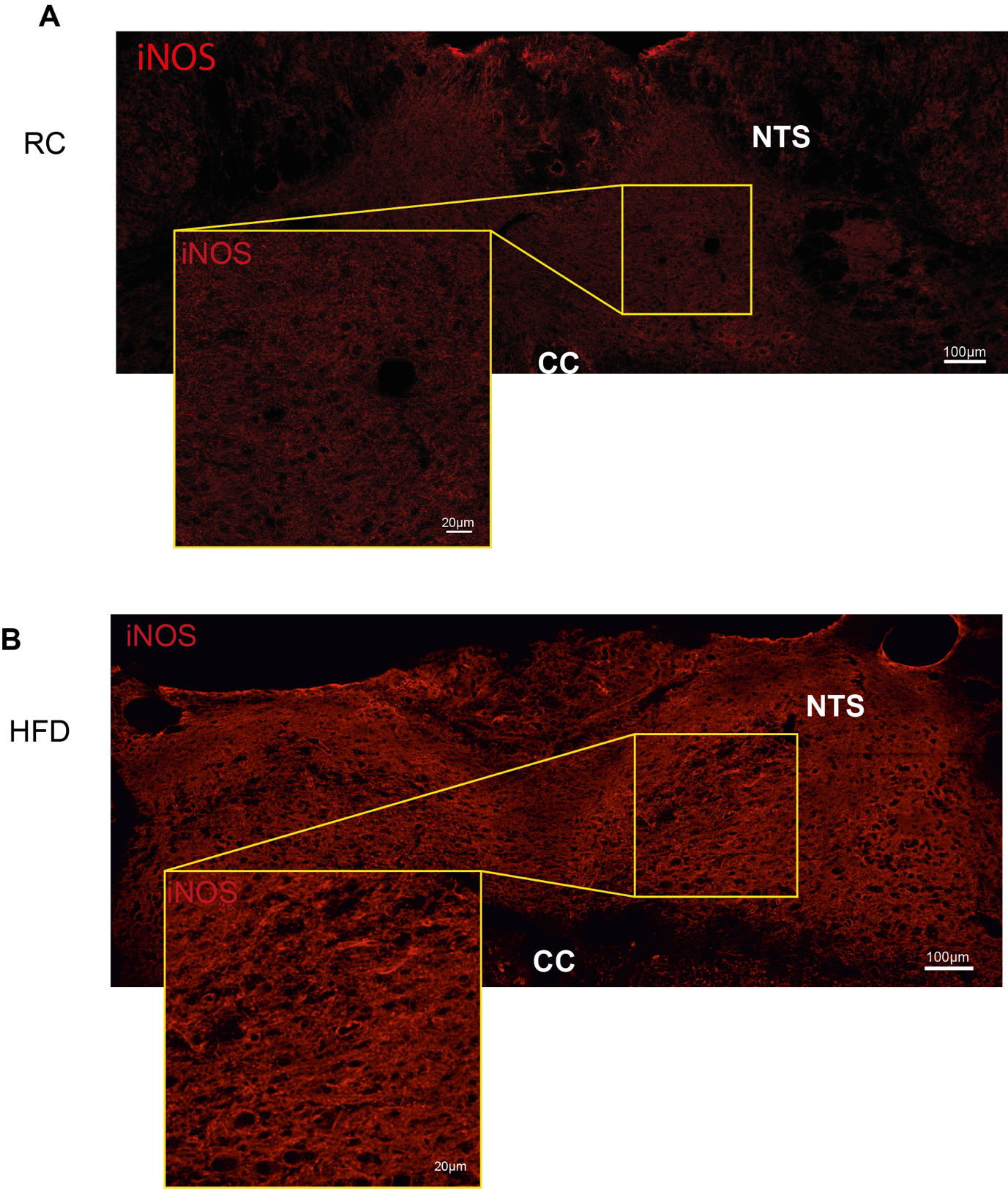
**

**Figure S3: HFD increases iNOS levels in the DVC.** Representative images of iNOS staining in the DVC of RC (**A**) and HFD-fed (**B**) rats. Image shows a large tile that includes a central canal and NTS of the DVC. A magnified area is also shown.

**
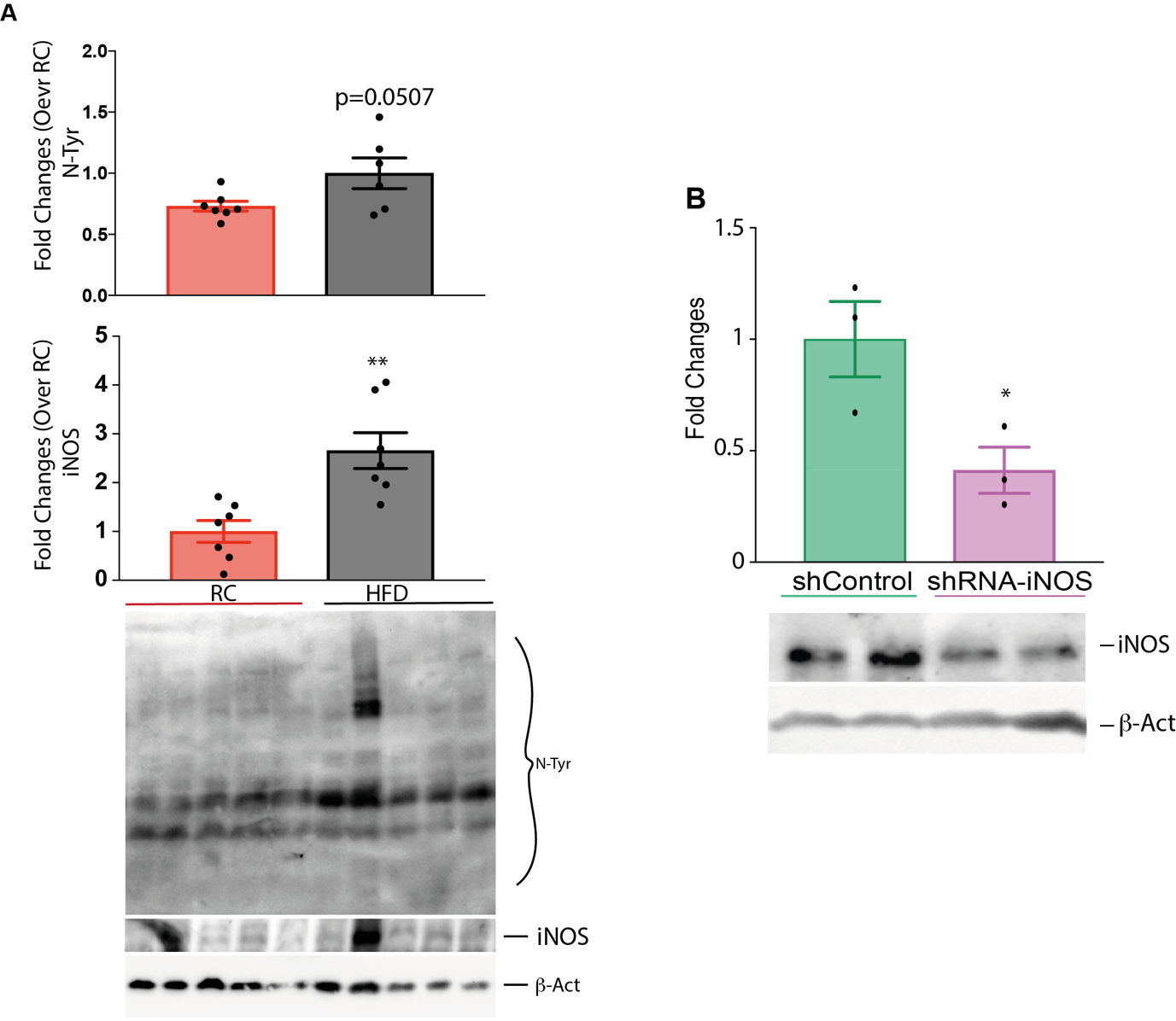
**

**Figure S4: HFD feeding increases iNOS and tyrosine nitration levels in the DVC and control blots for fig. 2 D. A**. Western blot analysis of the changes in iNOS or Nitryl Tyrosine (N-Tyr) levels in the DVC of HFD-fed rats compared with RC-fed rats. Representative western blot images of iNOS, N-Tyr, and β-actin are also shown. **B.** PC12 cells infected with a lentivirus expressing shRNA for iNOS or Control scramble virus were lysed and iNOS knockdown levels are quantified by western blotting. In the figure the quantification with a representative western blot is shown. All data are expressed as mean ± SEM *n*=3 for shControl and shiNOS-expressing cells. [*p < 0.05, **p <0.01]


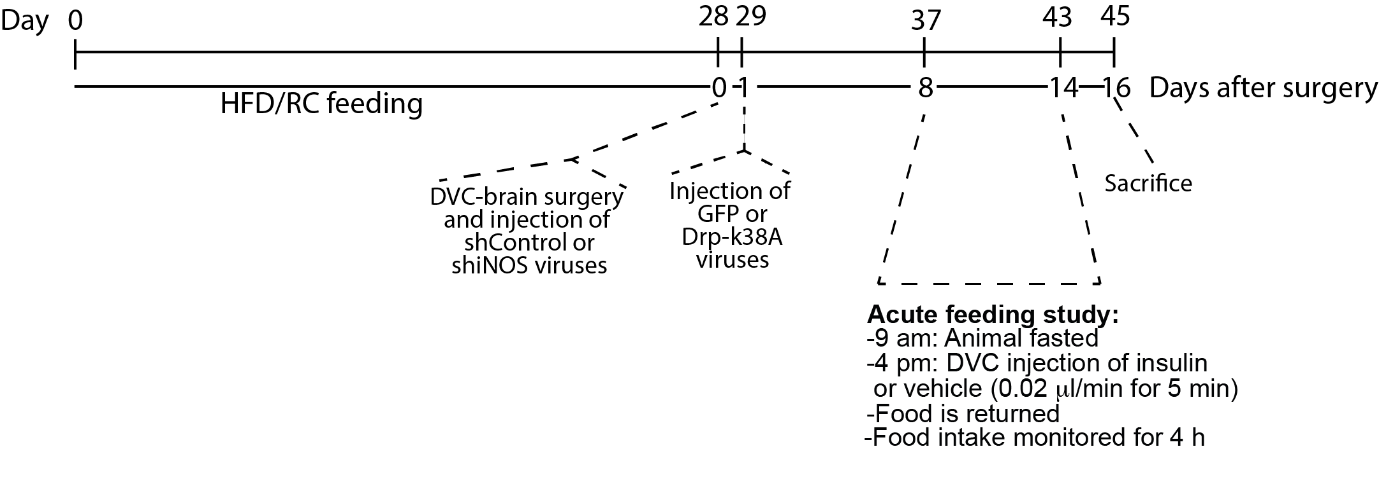


**Figure S5:** **Experimental design for the 28d HFD-fed obese model**. Rats were fed for 28 days with an HFD or control RC diet. On day 28, rats received DVC surgery**.** shControl and shiNOS virus were injected on surgery day; the GFP and Drp1-K38A viruses were injected on day 29. An acute feeding study was performed on days 8 and 14 after surgery.


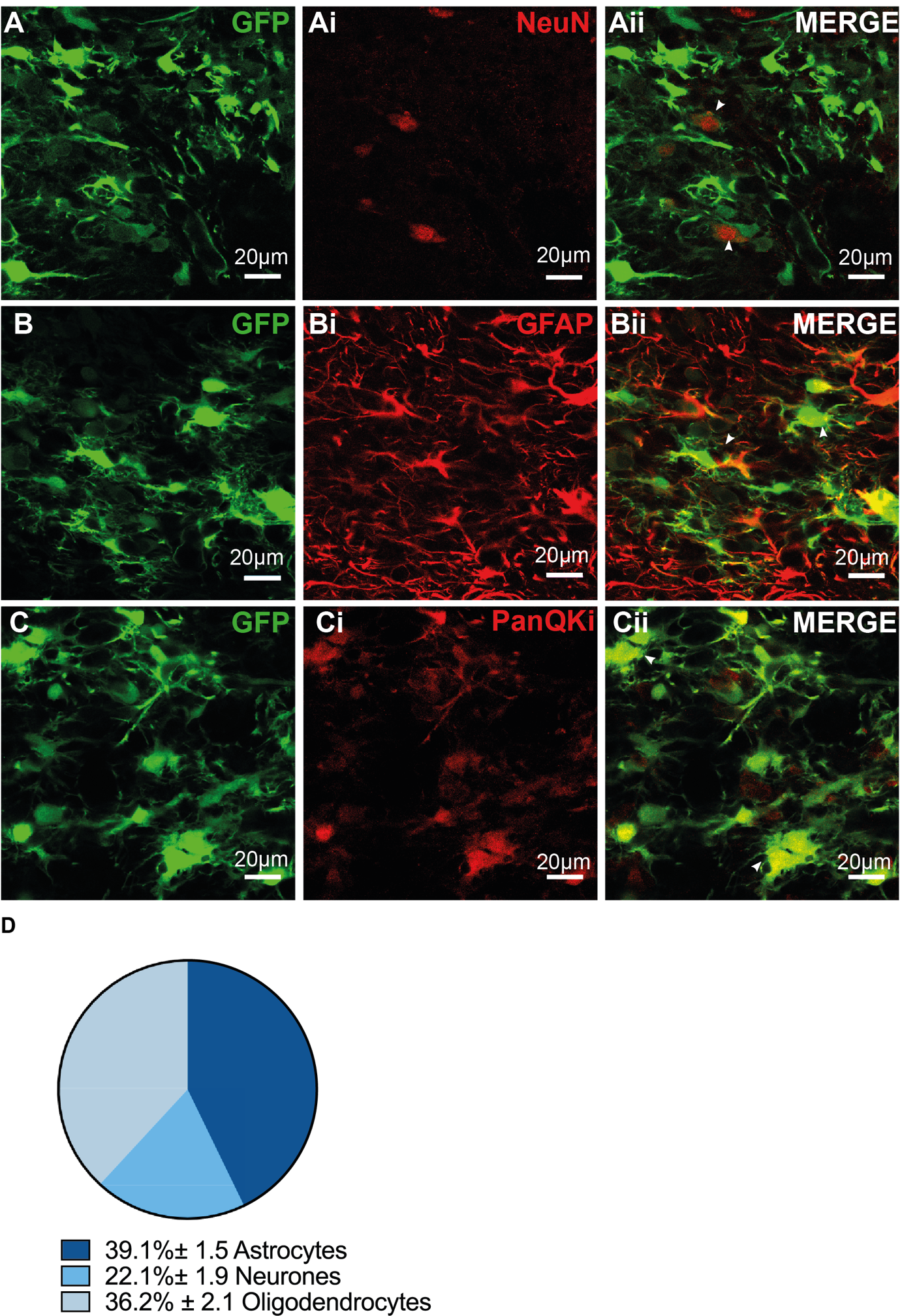


**Figure S6: GFP expression in neural cell types. (A-Aii)** Representative confocal images illustrating labelling of GFP expression in (A), NeuN (Ai), and dual labelling (Aii) in the DVC. **(B-Bii)** Representative confocal images illustrating labelling of GFP expression in (B), GFAP (Bi), and dual labelling (Bii) in the DVC. **(C-Cii)** Representative confocal images illustrating labelling of GFP expression in (C), PanQKi (Ci), and dual labelling (Cii) in the DVC. **(D)** Quantification of the co-localised cells.

Closed arrows denote co-localised cells. Images and quantification represent the average of n= 3 for NeuN and PanQKi, and n= 4 for GFAP animals. Three images were quantified per animal).

**
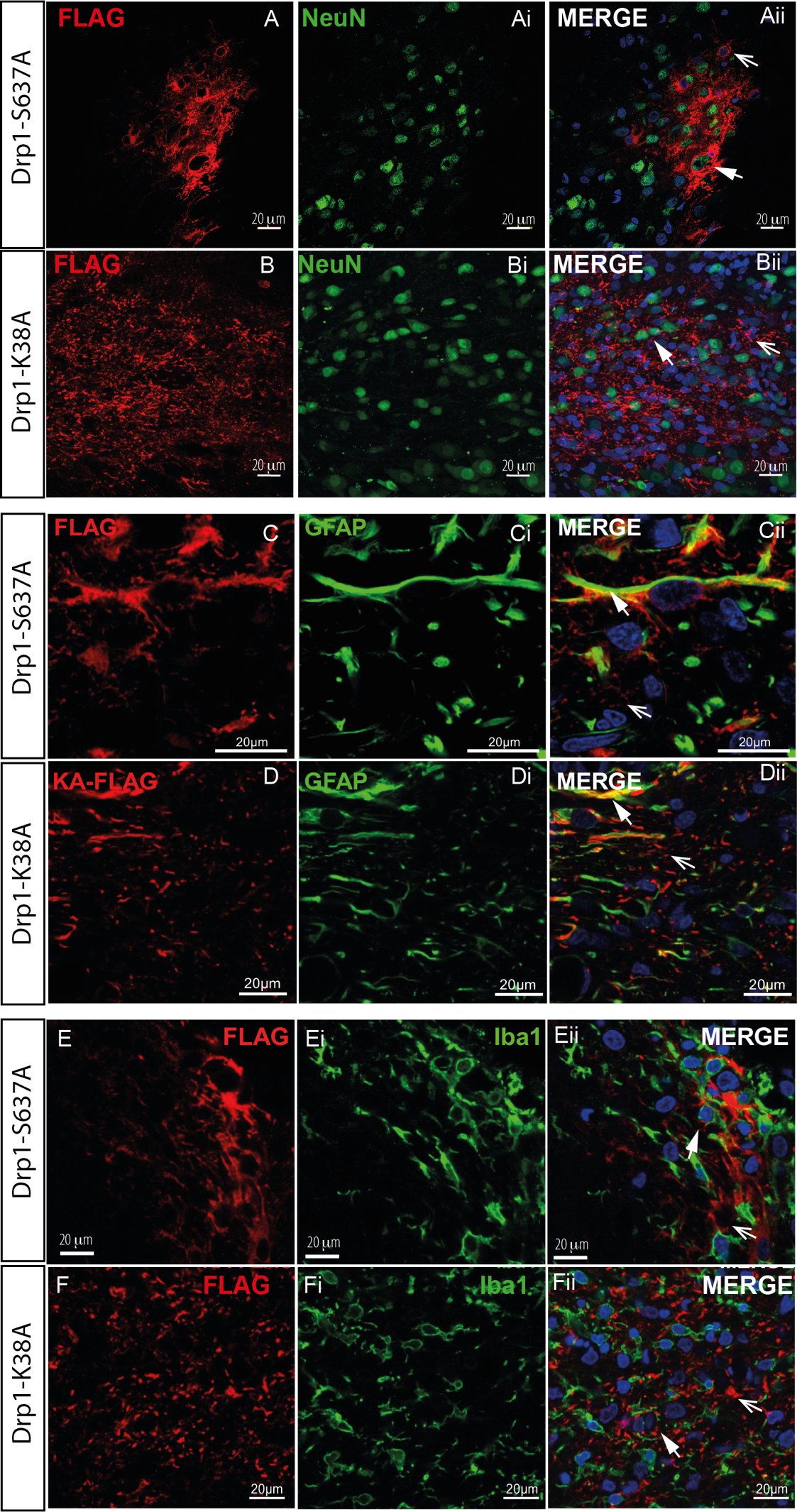
**

**Figure S7: Expression of the constitutively active form of Drp1, Drp1-S637A and the dominant negative form of Drp1, Dr1-K38A in neuronal cells.** **(A-Aii)** Representative confocal images illustrating the expression Drp1-S637A (A), NeuN (Ai), and dual labelling (Aii) in the DVC. Blue in Aii represents Nuclei stained with Dapi. **(B-Bii)** Representative maximal intensity confocal images illustrating the expression of Drp1-K38A (B), NeuN (Bi), and dual labelling plus Dapi in blue (Bii) in the DVC. **(C-Cii)** Representative confocal images illustrating the expression of Drp1-S637A (C), GFAP (Ci), and dual labelling plus Dapi in blue (Cii) in the DVC. **(D-Dii)** Representative confocal images illustrating the expression of Drp1-K38A (D), GFAP (Di), and dual labelling plus Dapi in blue (Dii) in the DVC. **(E-Eii)** Representative confocal images illustrating the expression of Drp1-S637A (E), Iba1 (Ei), and dual labelling plus Dapi in blue (Eii) in the DVC. **(F-Fii)** Representative confocal images illustrating the expression Drp1-K38A (F), Iba1 (Fi), and dual labelling plus Dapi in blue (Fii) in the DVC. Open arrows denote non-colocalised cells. Closed arrows denote colocalised cells.

**
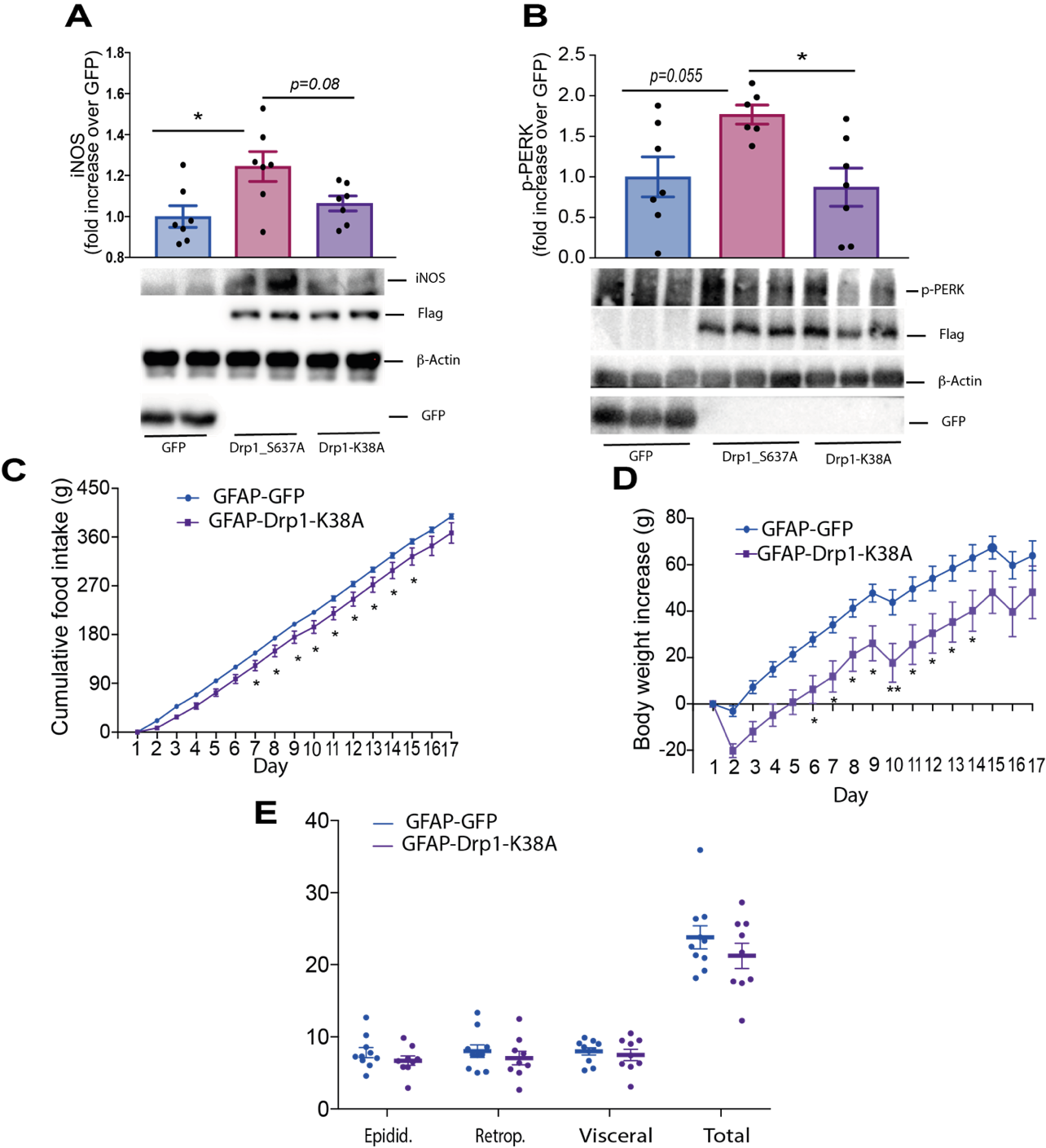
**

**Figure S8: Inhibition of mitochondrial fission in GFAP-expressing astrocytes decreases iNOS and p-PERK levels in HFD-fed rats; inhibition of mitochondria fission in astrocytes of the DVC decreases food intake, and body weight in regular chow-fed rats**. (**A-B**) Western blot analysis of iNOS (A) and p-PERK (B) levels in astrocytes cell expressing GFP, Drp1-S637A, and Drp1-K38A. Data are the average of n=7 samples for each condition. Representative western blot images show GFP, Flag-tagged Drp1 mutants, β-actin, iNOS, and p-PERK expression. **(C to E)** A double cannula was inserted into the NTS of the DVC in RC-fed rats on day 0. On day 1, rats were injected with an adenovirus expressing either GFAP-GFP or GFAP-Drp1K38 in the NTS of the DVC. Food intake and body weight were measured daily for 17 days. (**C**) Cumulative food intake from day 1. (**D**) Body weight increase from day 1. (**E**) White adipose tissue measurements- epididymal, retroperitoneal, and visceral fat collected on the day of sacrifice. Data are shown min ± SEM, with each single point highlighted. Data are representative of n=8 rats for both GFP and Drp1-K38A. *p < 0.05, **p <0.01
